# Supplementary figures and images for: Generative and interpretable machine learning for aptamer design and analysis of in vitro sequence selection
Source: PLoS Comput Biol. 2022 Sep 29;18(9):e1010561. doi: 10.1371/journal.pcbi.1010561 (PMC9553063; doi:10.1371/journal.pcbi.1010561)

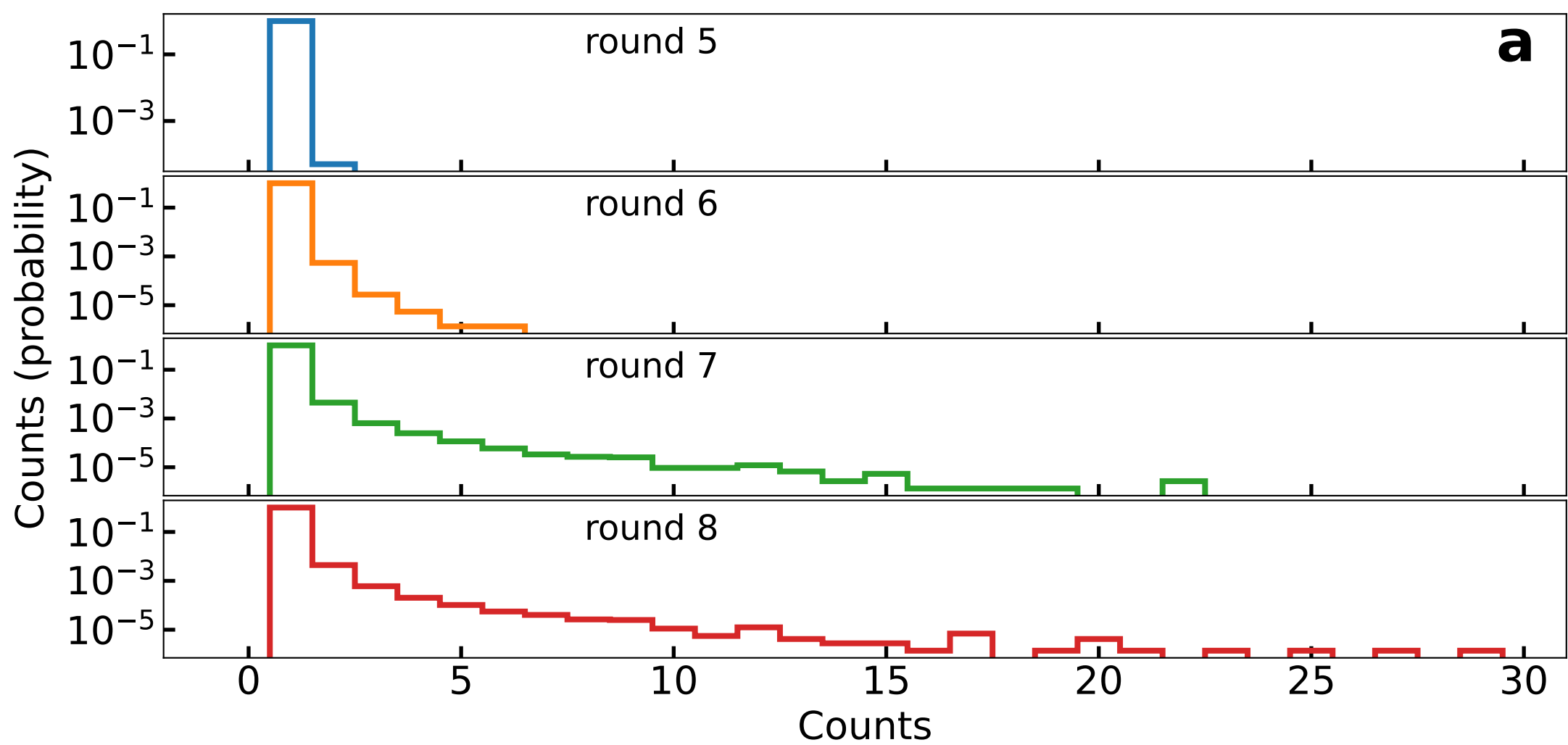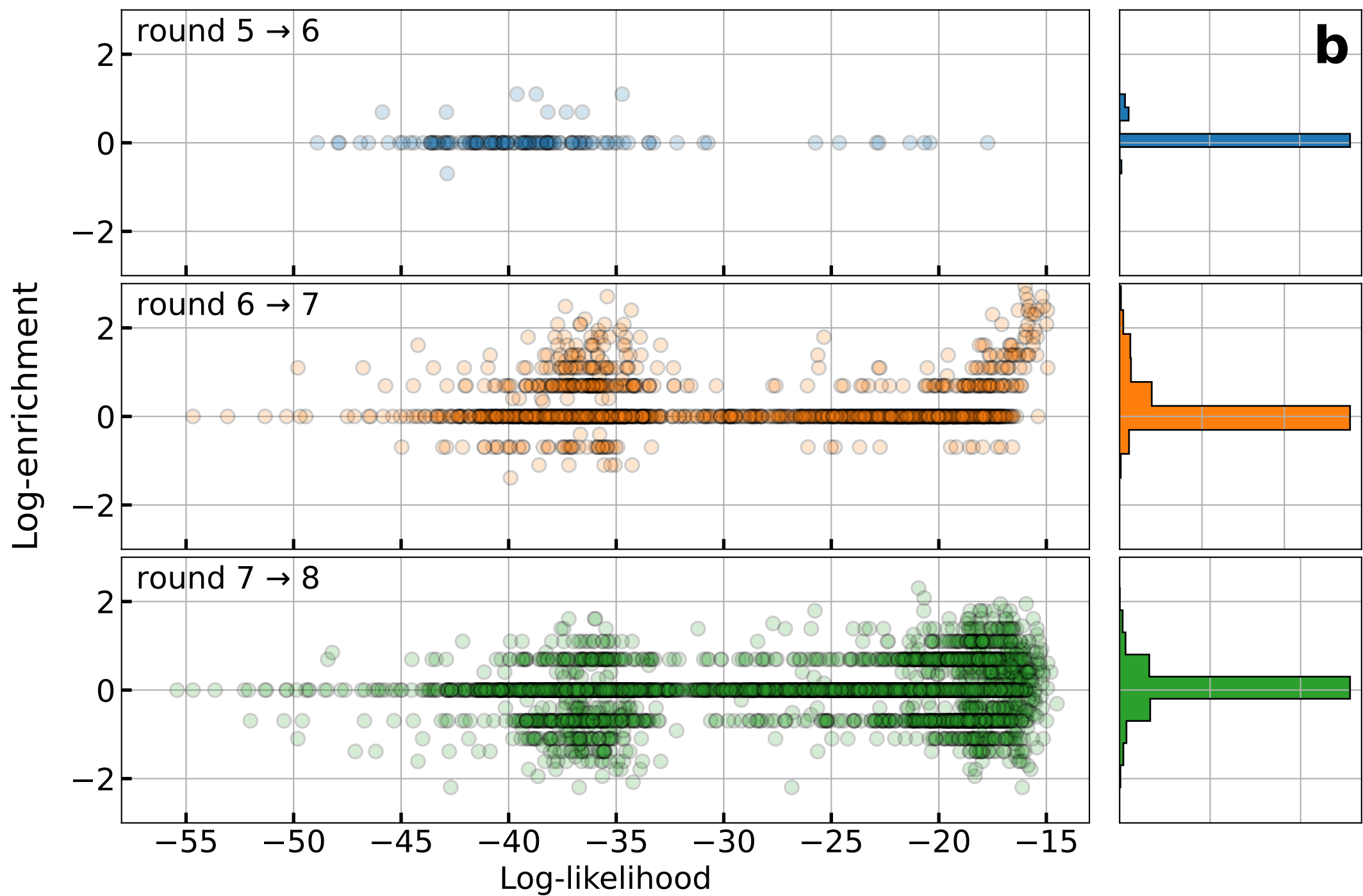

Supplement: S1 Fig — Panel A: probability density function of the counts observed for the double aptamers in each round. Notice the log scale on the y axis. Panel B: for each pair of consecutive rounds, we plot here the logarithm of the ratio of counts of the sequences present in both rounds (left) and the corresponding histogram (right), against the log-likelihood of the sequence computed with the RBM-DC model. (PDF) [file pcbi.1010561.s001.pdf]

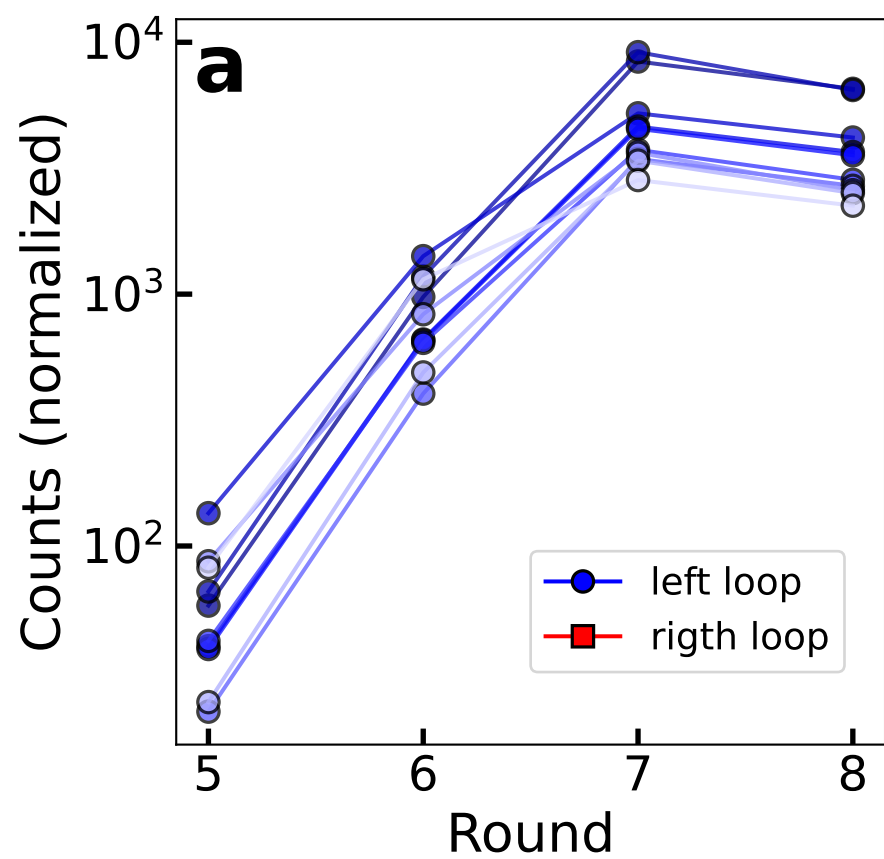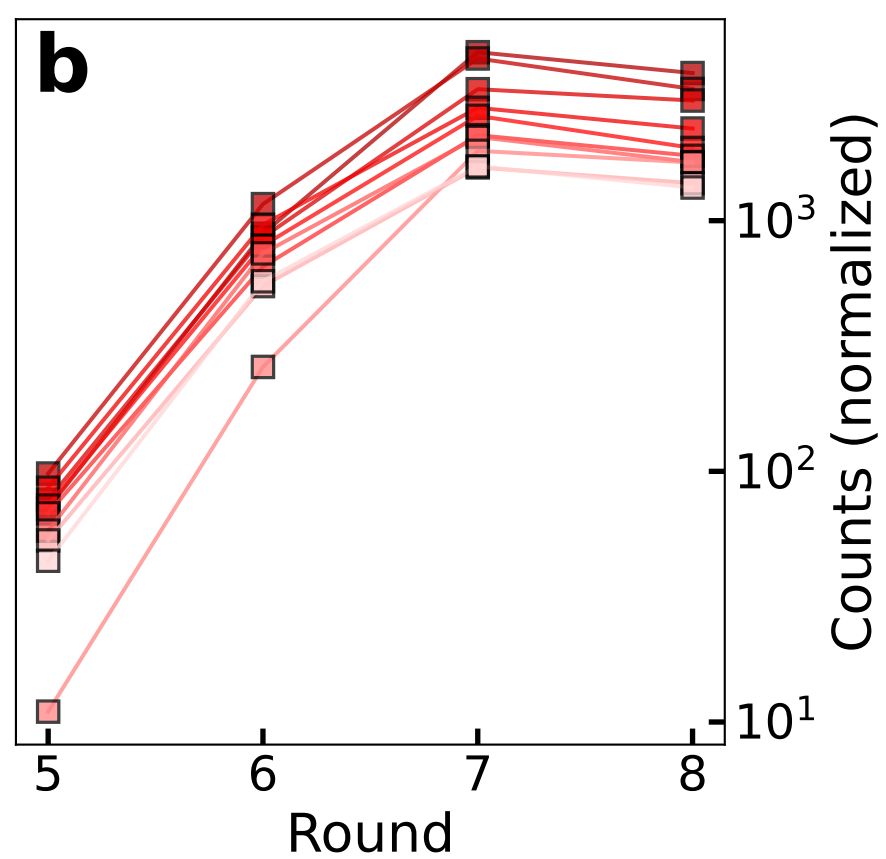

Supplement: S2 Fig — Counts have been re-scaled by a factor so that the total number of counts in each round is constant. (PDF) [file pcbi.1010561.s002.pdf]

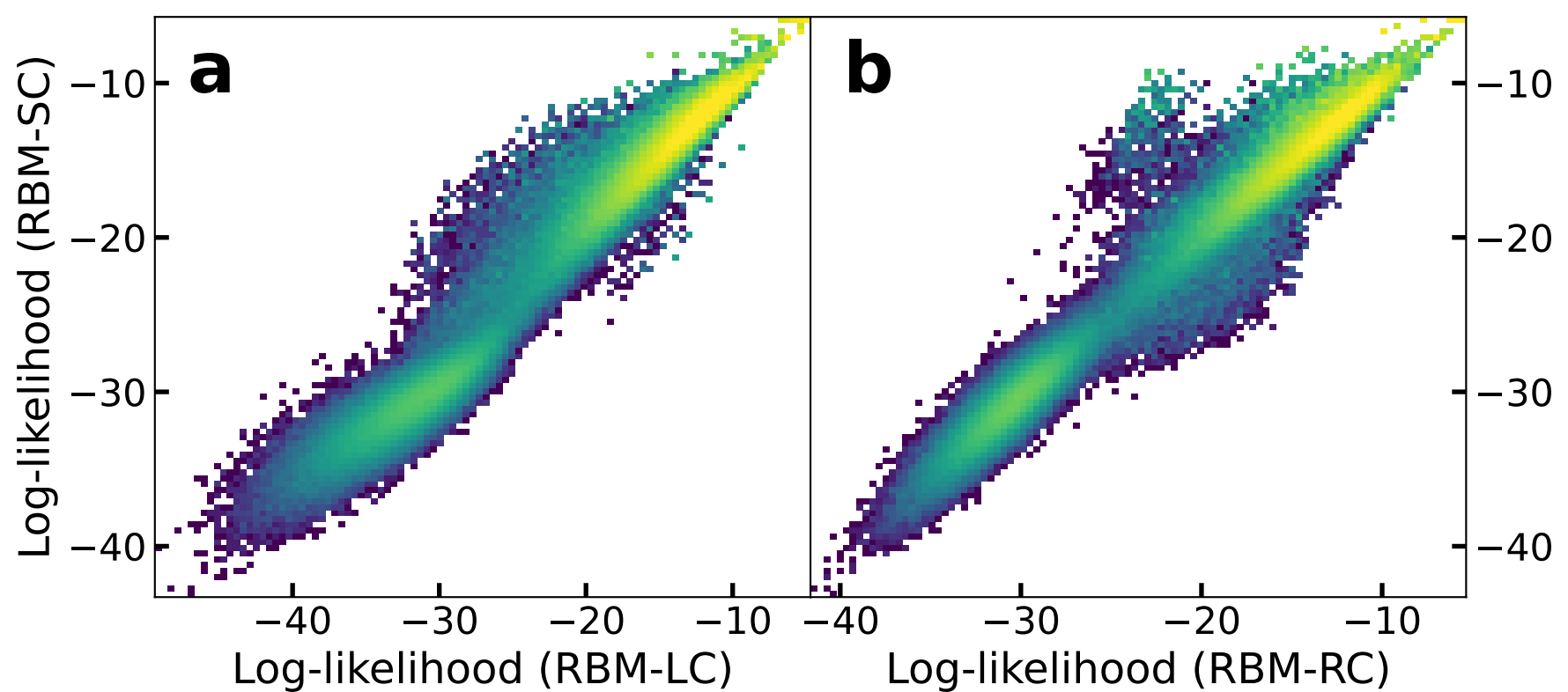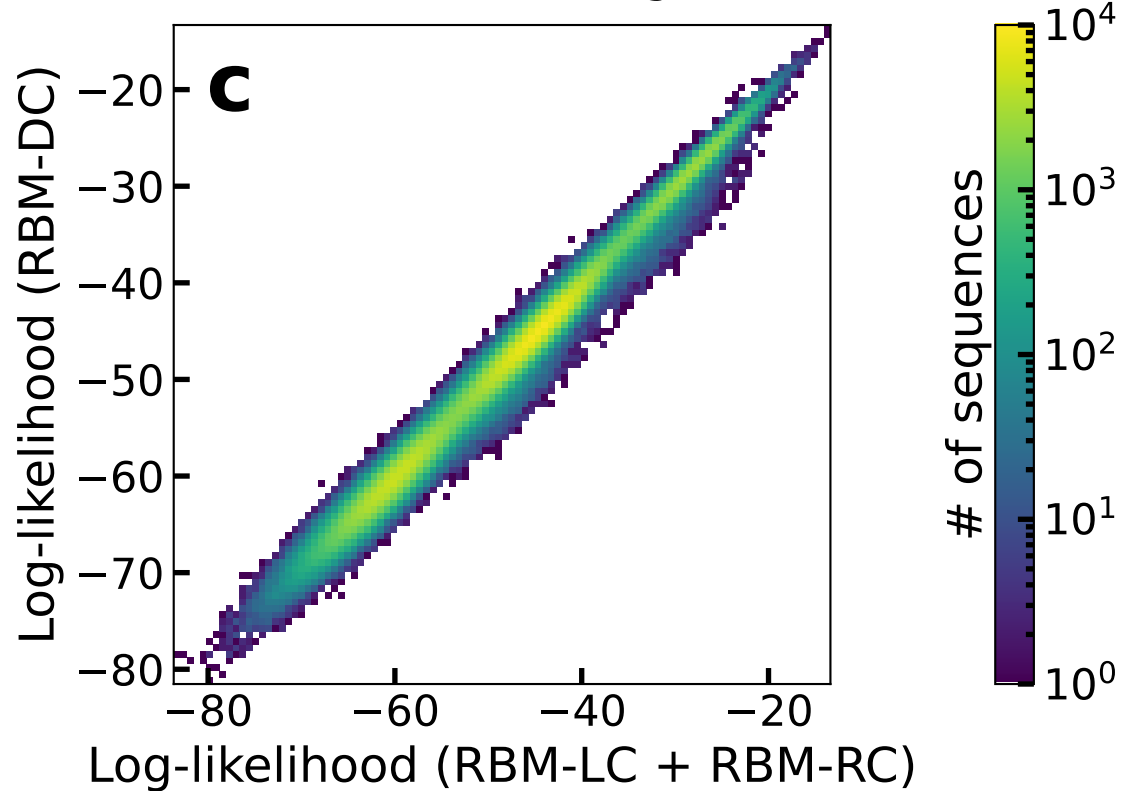

Supplement: S3 Fig — Log-likelihood computed with the RBM-SC model and with the RBM-LC model (trained on left single-loop sequences at round 8, see S1 Appendix) in panel A or RBM-RC model (trained on right single-loop sequences at round 8, see S1 Appendix) in panel B for the single-loop sequences observed at round 8. The slope and the R2 values of the linear fit are respectively 0.96 and 0.98 for panel A, and 1.05 and 0.97 for panel B. Panel C: log-likelihood computed with the RBM-DC model for the double-loop sequences observed at round 5, compared with the sum of the log-likelihood obtained by using RBM-LC to score the left loop and RBM-RC to score the right loop. The slope and the R2 value of the linear fit are, respectively, 0.99 and 0.99. (PDF) [file pcbi.1010561.s003.pdf]

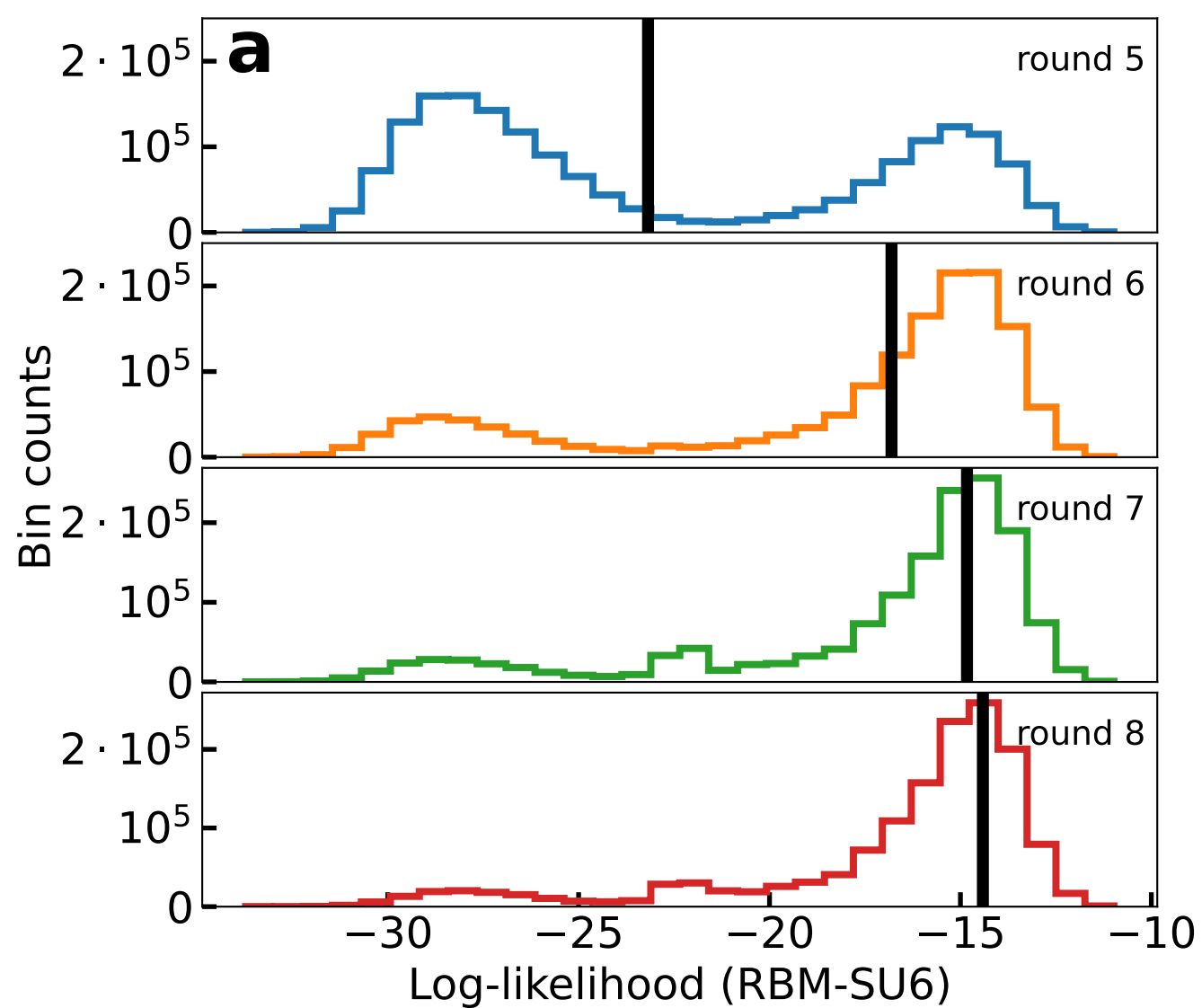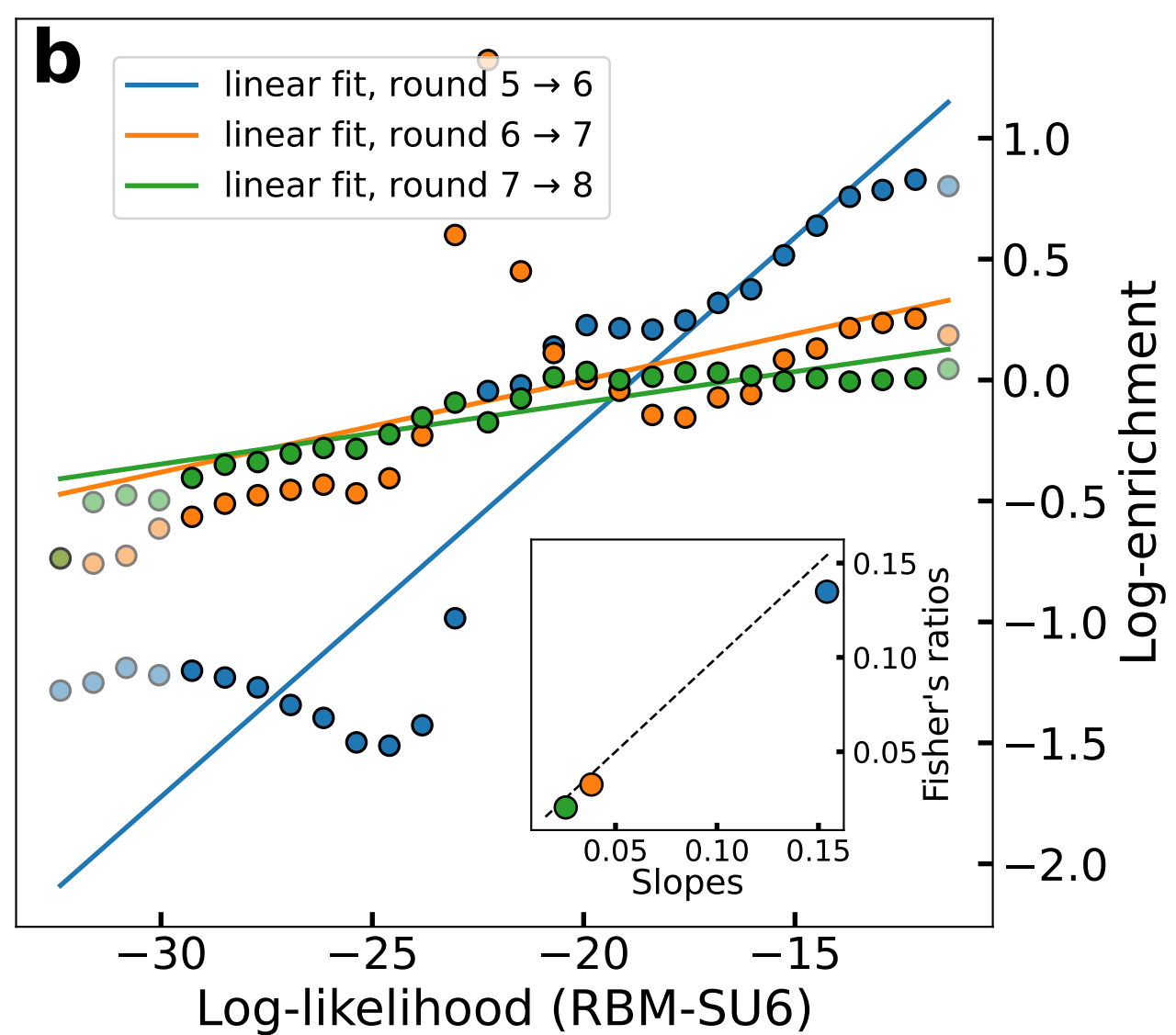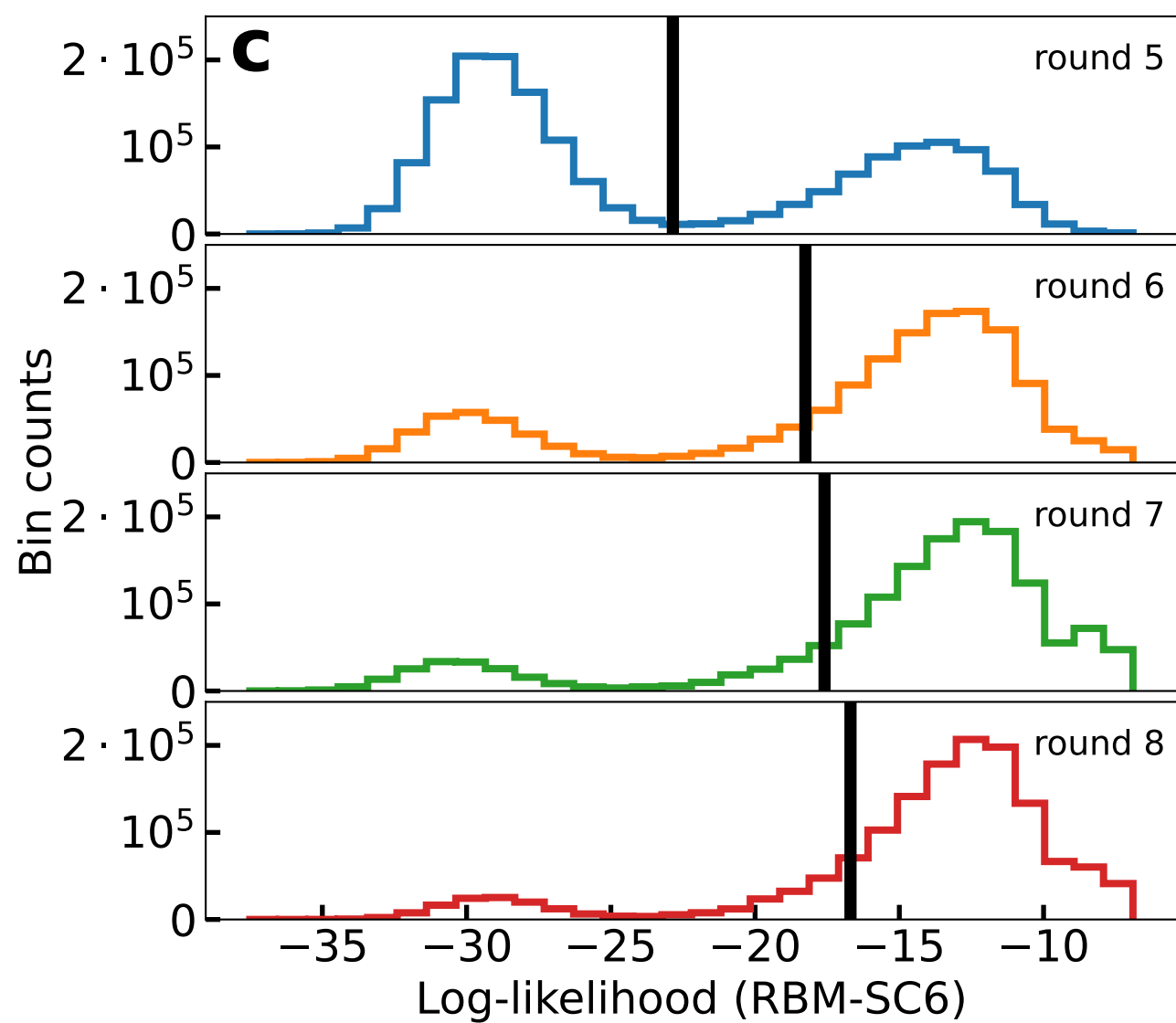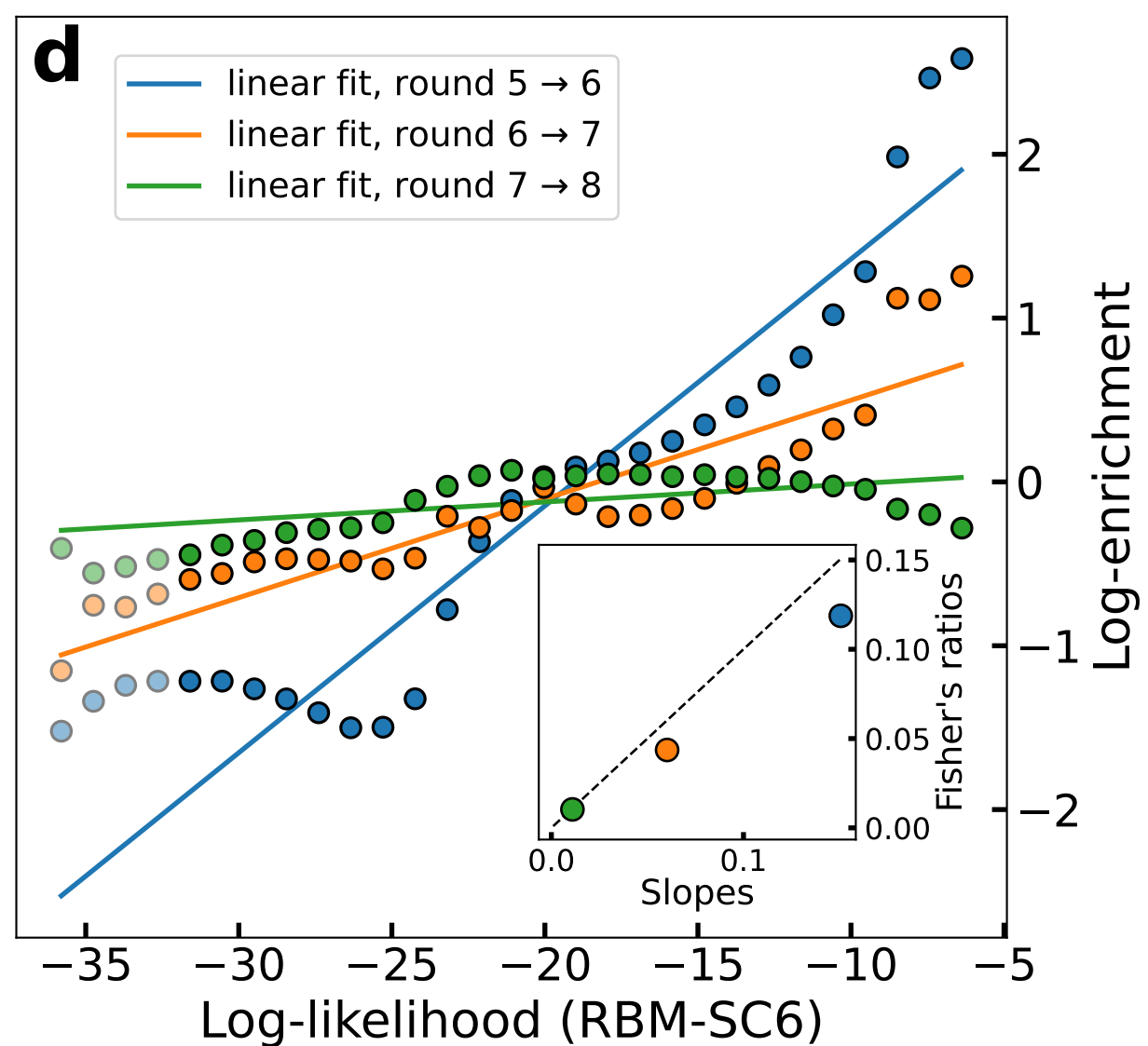

Supplement: S4 Fig — Panels A, C show the histograms of log-likelihoods at each round, as computed by RBM-SU6 (panel A) and RBM-SC6 (panel C). Panels B, D show the scatter plot of log-enrichment of each bin in the left panels, and the corresponding log-likelihood. In the inset, the slope of each linear fit appearing in the main plot is compared with the same quantity estimated as a Fisher’s ratio (see Sec RBM’s log-likelihood is an accurate predictor of the aptamer’s fitness). The dashed black line is the x = y line. (PDF) [file pcbi.1010561.s004.pdf]

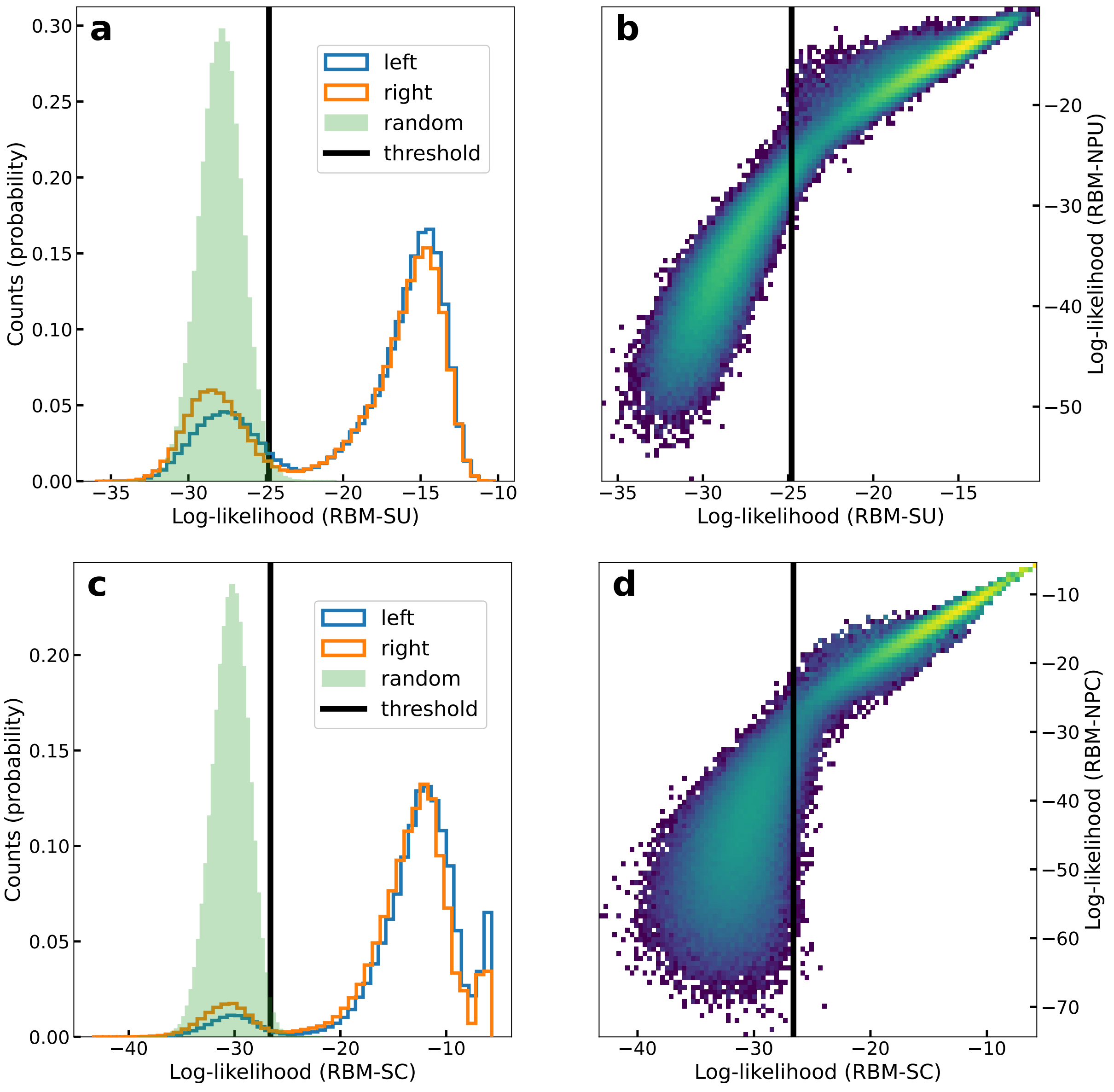

Supplement: S5 Fig — Left side: histograms of log-likelihoods of left (blue) and right (orange) loops computed with RBM-SU (panel A) or RBM-SC (panel C) for sequences observed in round 8 (unique in panel A, with their counts in panel B), together with that of 5 ⋅ 105 random uniform sequences (light green); the black line is the 99-quantile of the light green histogram, and parasite sequences are defined as those which have lower log-likelihood than the black line, while at the same time the other loop of the 40-nt aptamer has log-likelihood larger than the threshold. Right side: log-likelihood of the RBM trained after excluding parasite sequences at round 8 (RBM-NPU for panel B, RBM-NPC for panel D) versus that of the RBM-SU (panel B) or RBM-SC (panel D) model. A linear fit for the points at the right-hand side of the black line (which is the same of panels a for panel B, and of panel C for panel D) gives a slope of 1.0 and a R2 of 0.92 for panel B, and a slope of 1.0 and a R2 of 0.96 for panel D. For points at the left-hand side of the black line the slope is 2.6 with an R2 of 0.79 for panel B, and the slope is 2.0 with an R2 of 0.33 for panel D. (PNG) [file pcbi.1010561.s005.png]

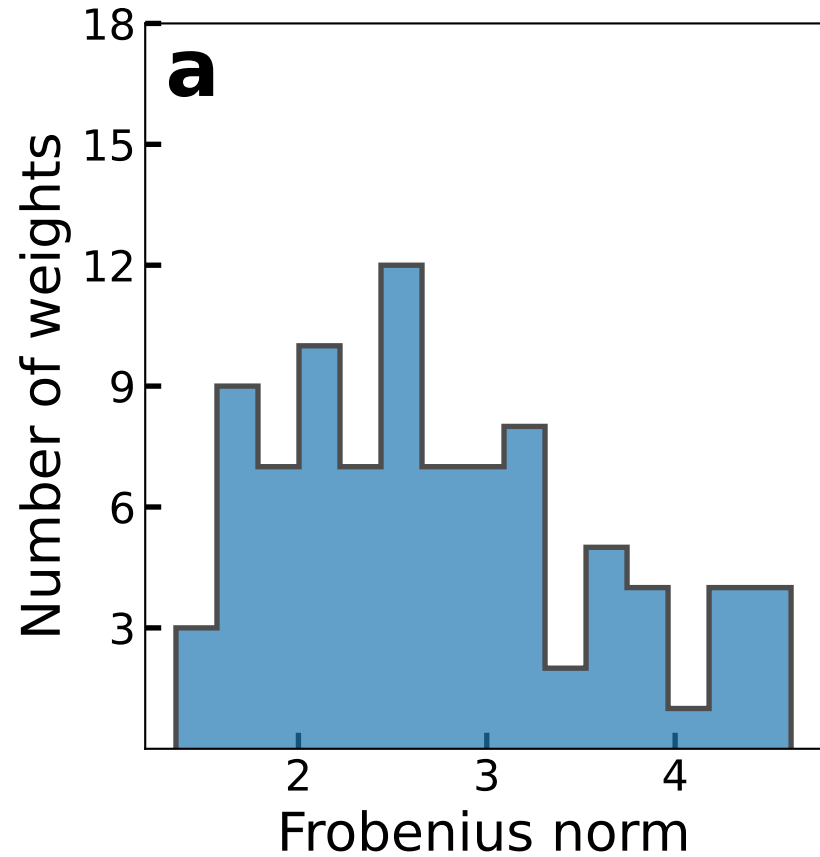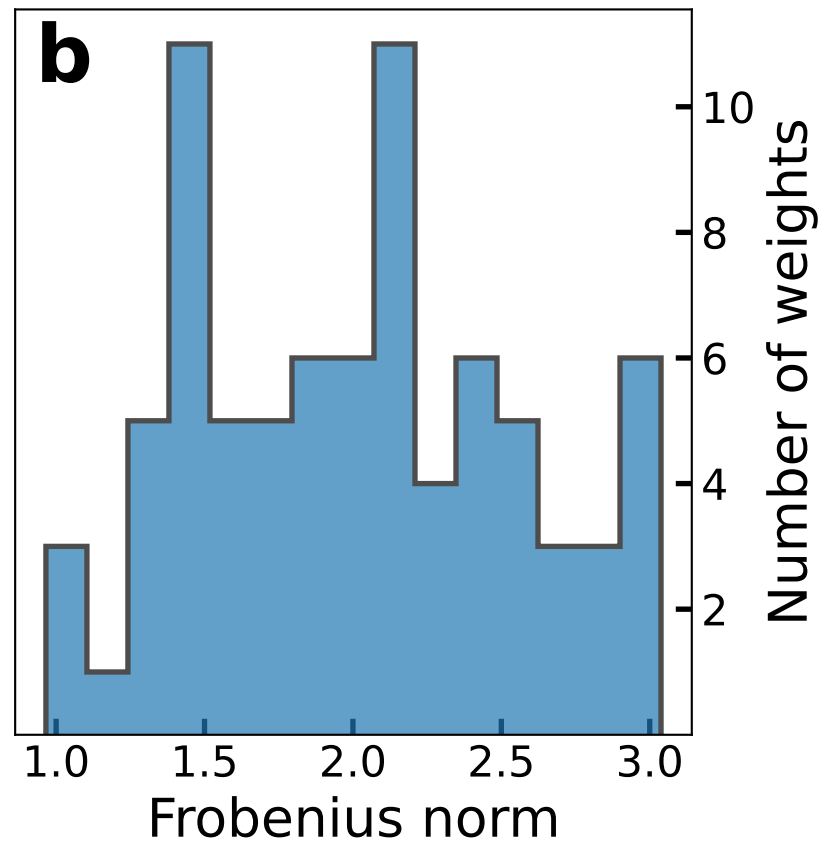

Supplement: S6 Fig — Panel A: Frobenius norms of the weights for RBM-DC. The logos corresponding to the 3 weights with largest Frobenius norm are given in Fig 4A–4C. Panel B: Frobenius norms of the weights for RBM-SC. The logos corresponding to the weight with the 2nd largest Frobenius norm and the one with the 7th largest Frobenius norm are given in Fig 4E and 4F. (PDF) [file pcbi.1010561.s006.pdf]

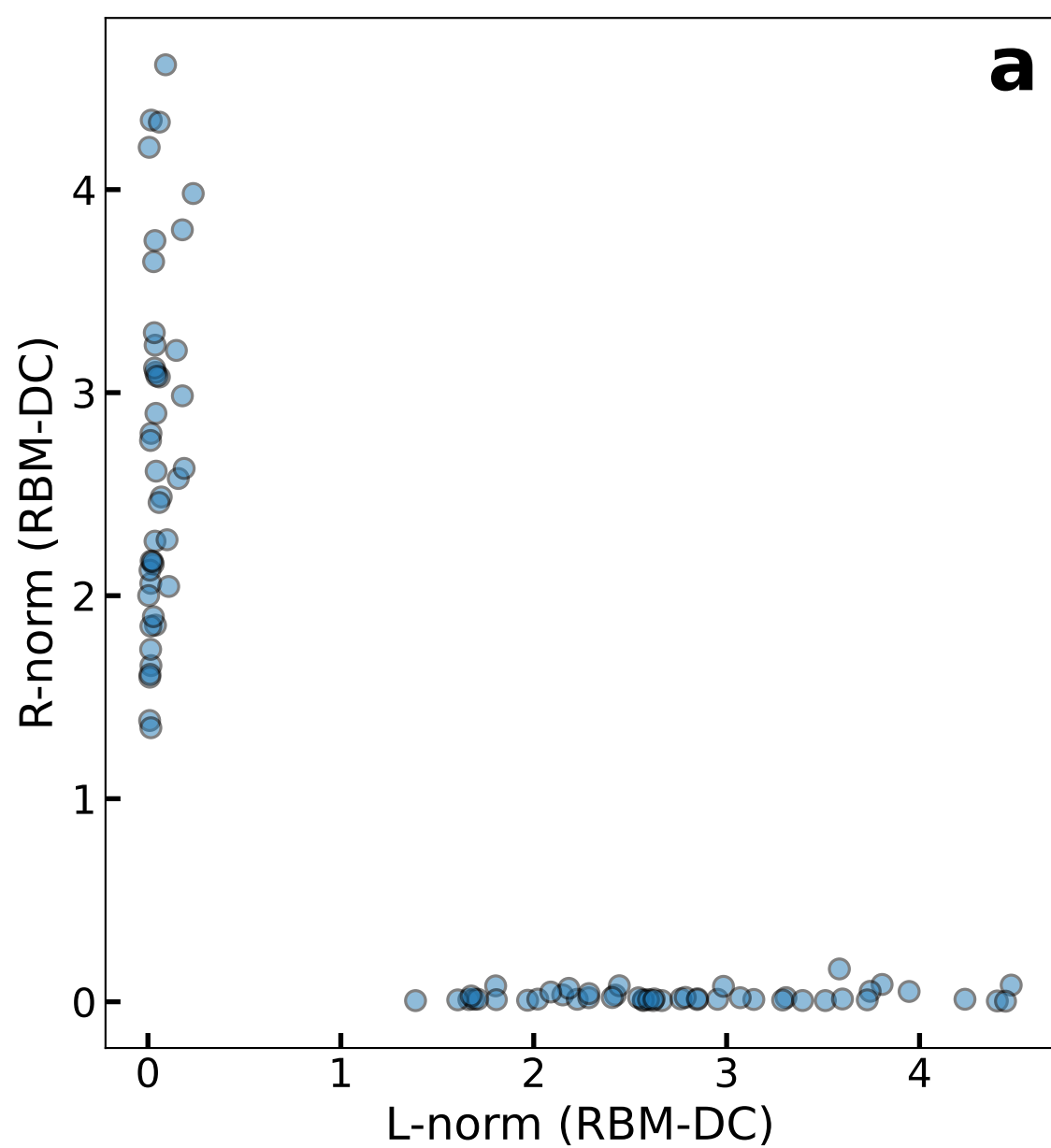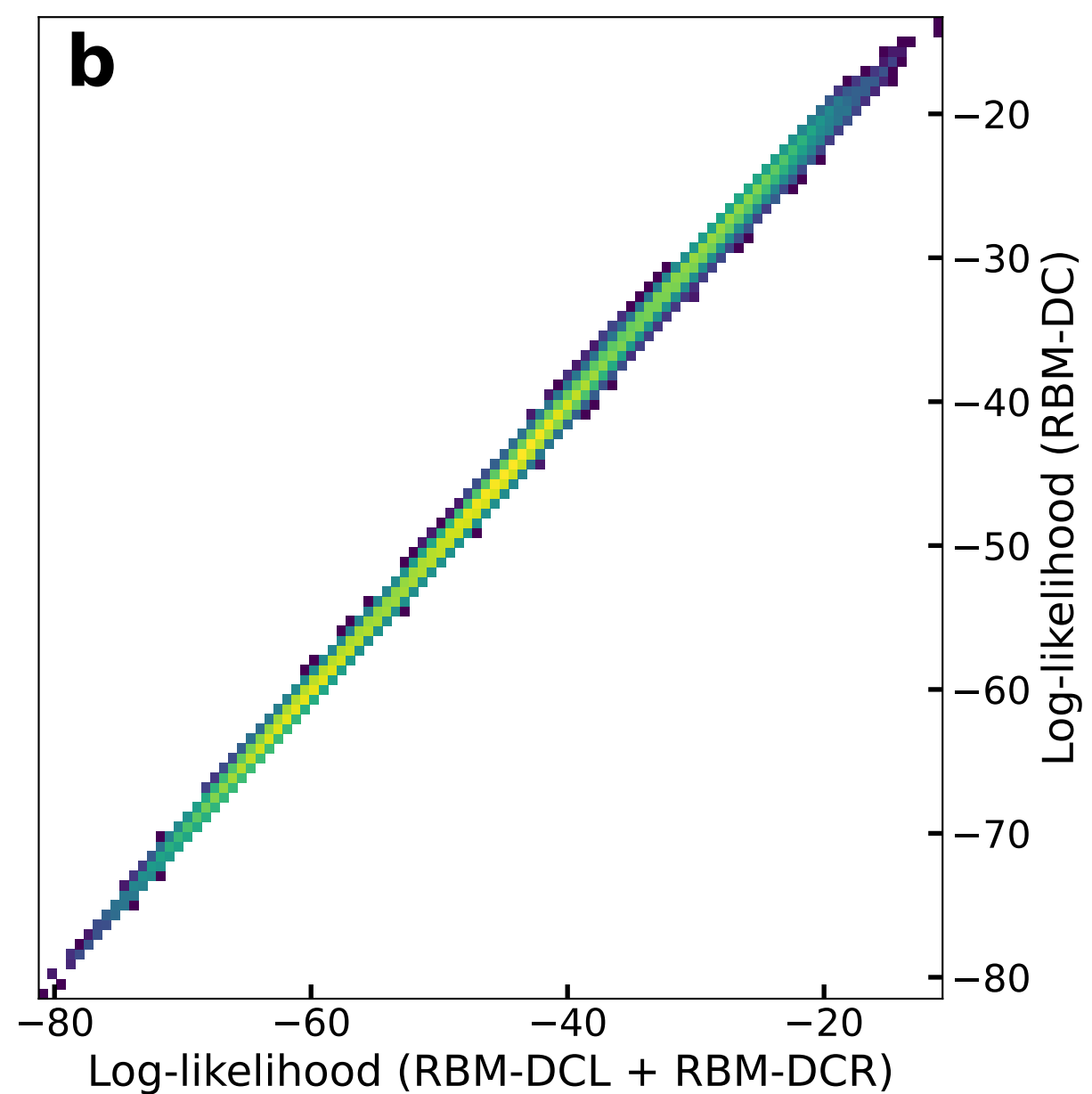

Supplement: S7 Fig — Panel A: Frobenius norms obtained for each weight of RBM-DC computed using only the first 20 visible units (L-norm in the x axis) or the last 20 visible units (R-norm in the y axis). Panel B: RBM-DCL and RBM-DCR are two RBMs with 20 visible units used to score left and right loops. RBM-DCL (RBM-DCR) is obtained from RBM-DC by using only its first (last) 20 visible units and their fields, and the hidden units with L-norm > R-norm (R-norm > L-norm) with their potentials, ignoring their interactions with the last (first) 20 visible units. In this panel, we compare, for each unique double-loop sequence observed at round 5, the log-likelihood of the RBM-DC model with the sum of the log-likelihoods obtained by using RBM-DCL to score the left loop and RBM-DCR to score the right loop. The slope of the linear fit is 0.99 and the R2 score is >0.99. (PDF) [file pcbi.1010561.s007.pdf]

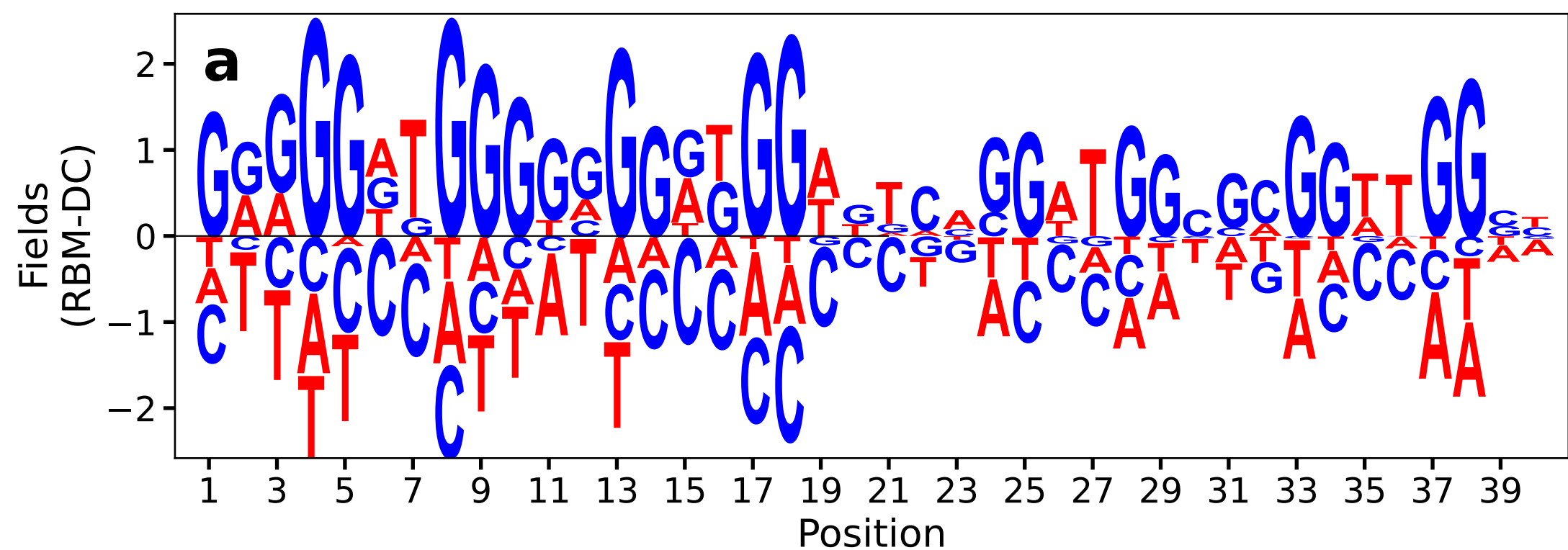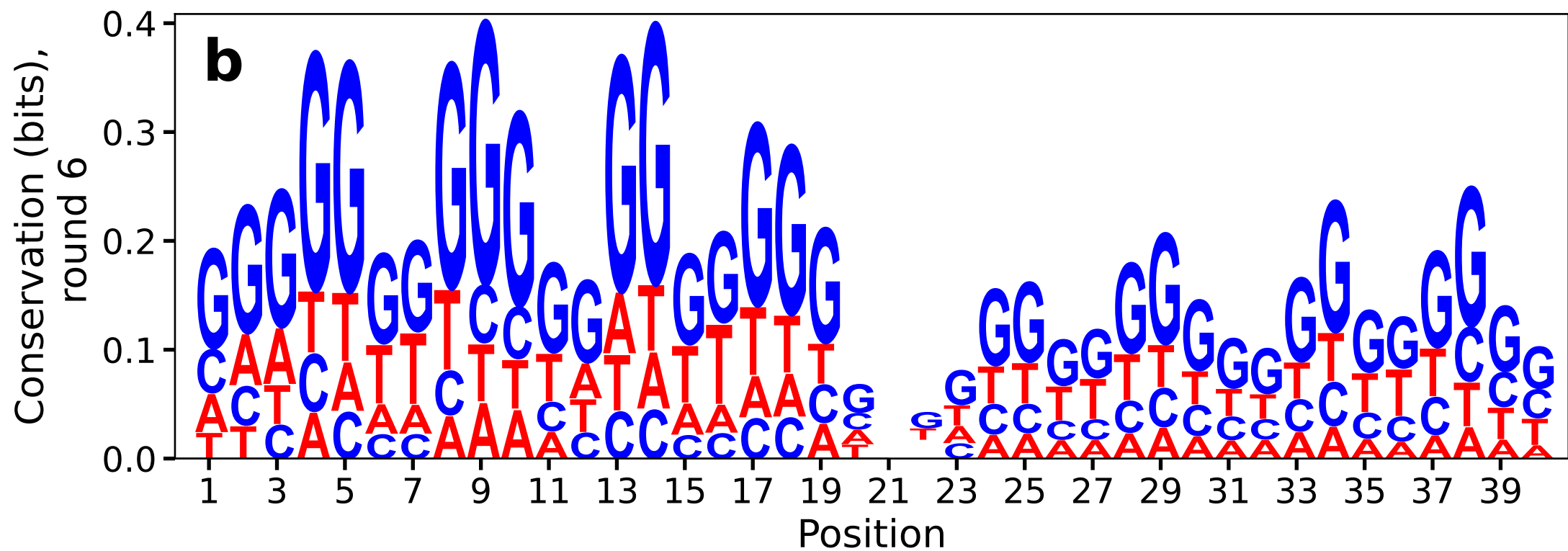

Supplement: S8 Fig — (PDF) [file pcbi.1010561.s008.pdf]

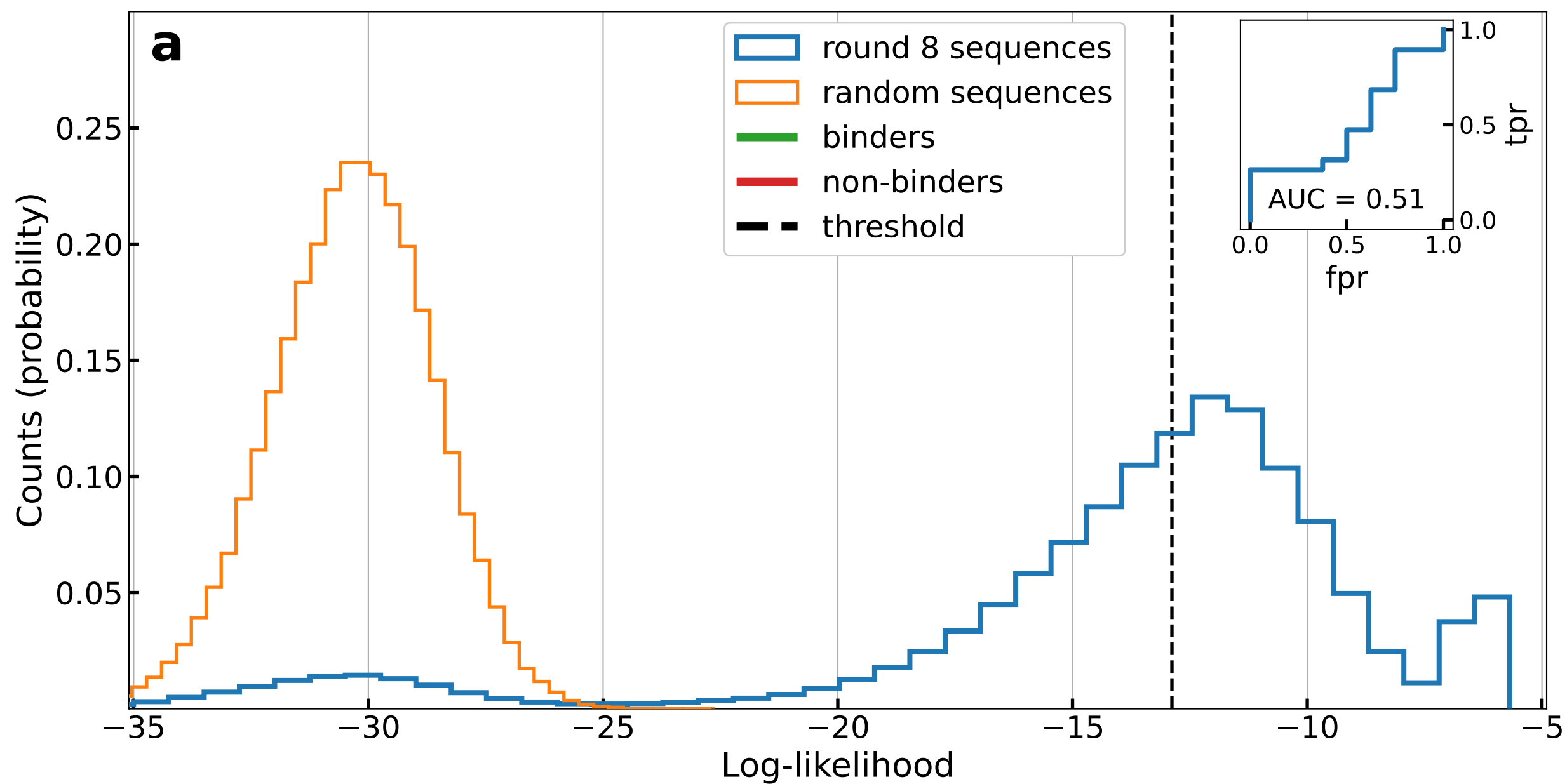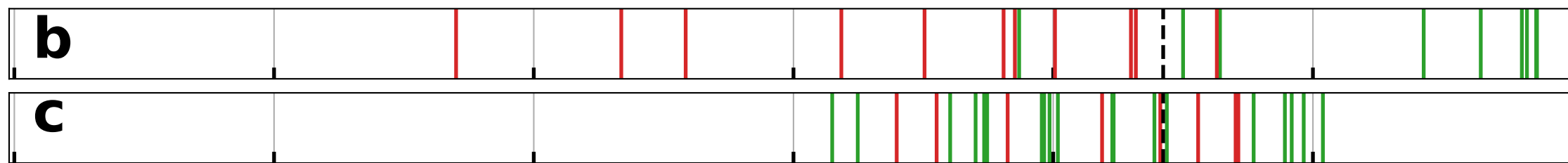

Supplement: S9 Fig — Panel A: Histogram of the log-likelihoods of all unique aptamers observed in the last round (blue line) and of uniformly random sequences (orange line), computed with RBM-SC trained on single-loop sequences from round 8, keeping information about the counts. Inset: AUC computed on the sequences generated by the RBM-SU model (panel C). Panel B: Vertical lines locate the log-likelihoods of sequences experimentally validated to be binders (green) or non binders (red). Sequences taken from a preliminary set described in S1 Table. Results allows us to determine the binding/non binding threshold, shown with the black dashed line. Panel C: same as panel B for sequences designed with the RBM-SU model, as described in Sec RBM trained from unique sequences generate diverse aptamers capable of binding thrombin (see Table 1). (PDF) [file pcbi.1010561.s009.pdf]

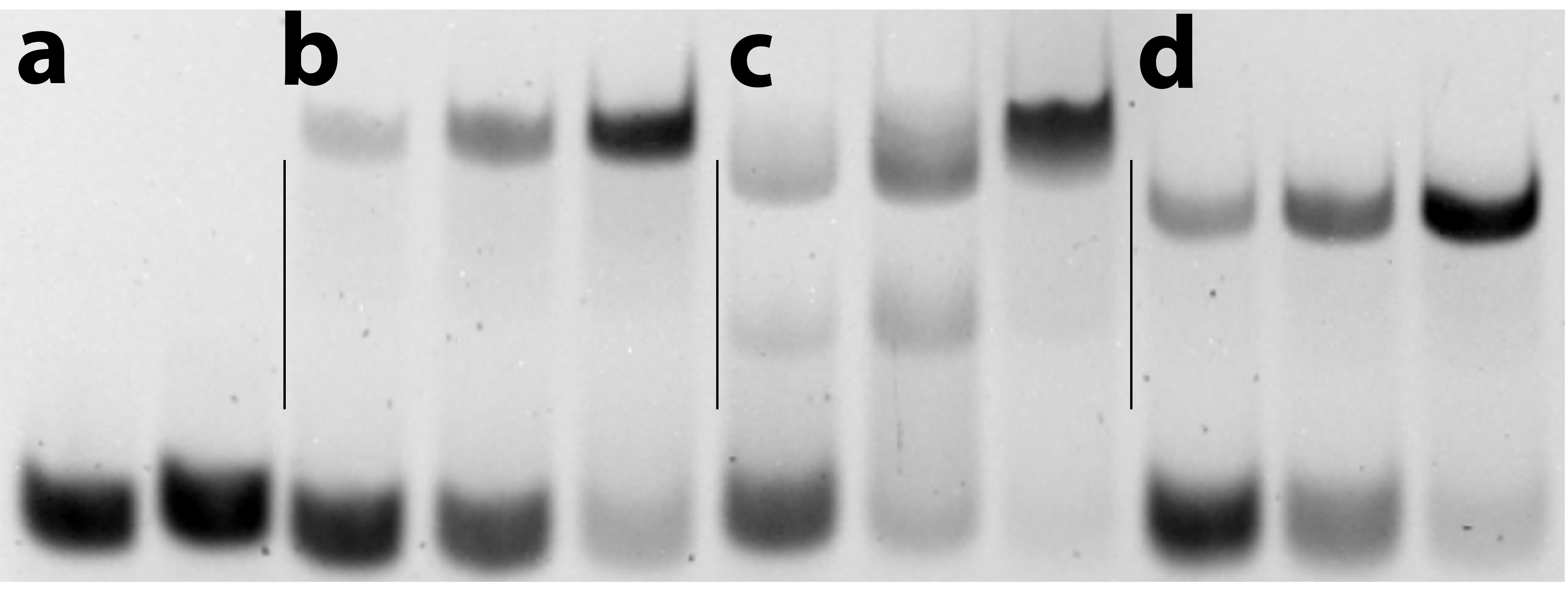

Supplement: S10 Fig — Panel A: lane 1 contains 5’ 6FAM labeled control sequence ThA, lane 2 contains 5’ 6FAM labeled control sequence ThD. Panel B: ThA mixed in varying ratios with Thrombin (1:0.32, 1:0.64, 1:1.08). Panel C: ThD mixed in varying ratios with Thrombin (1:0.32, 1:0.64, 1:1.08). Panel D: ThA + ThD mixed in varying ratios with Thrombin (1:1:0.32, 1:1:0.64, 1:1:1.08). (PNG) [file pcbi.1010561.s010.png]

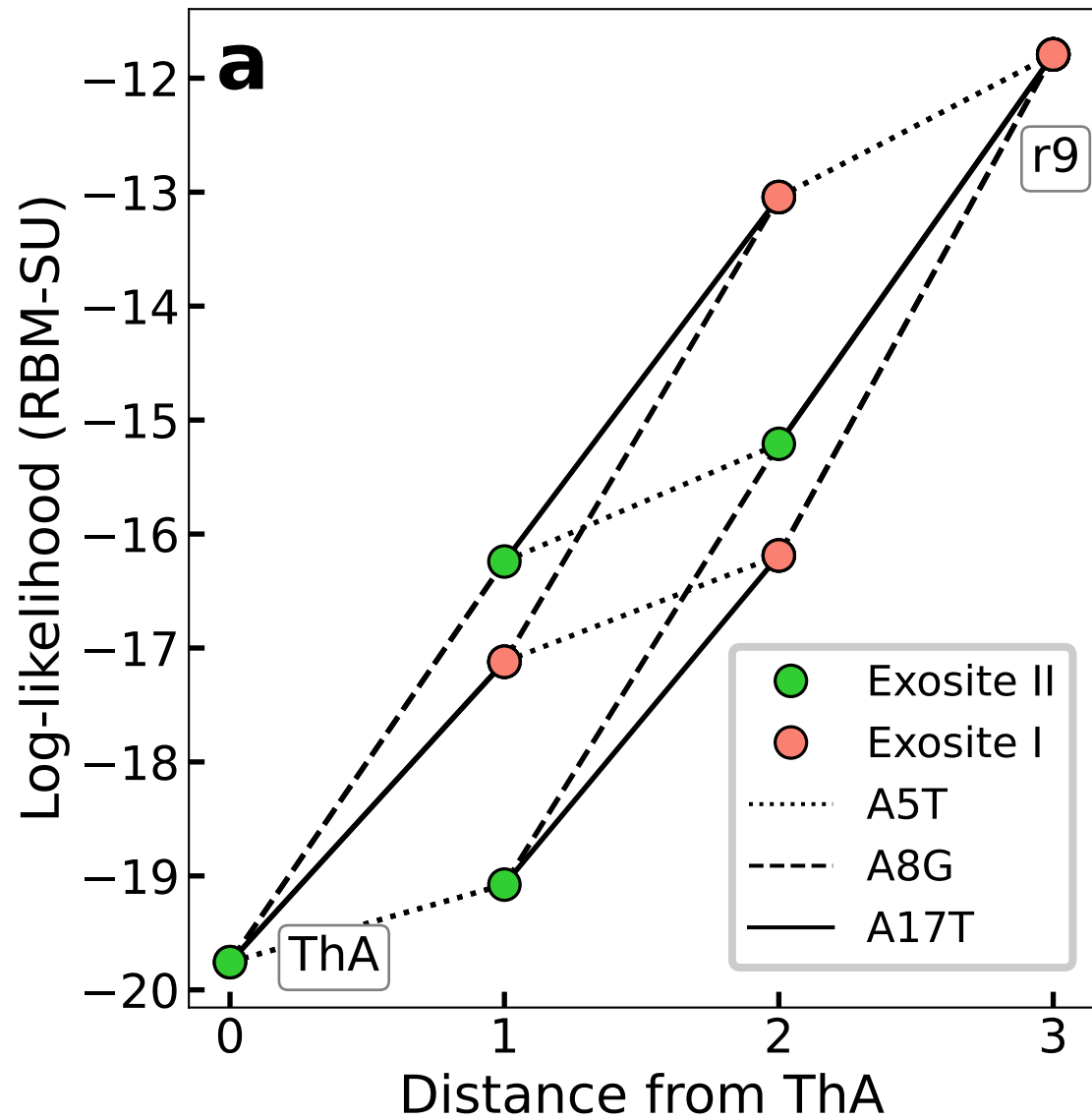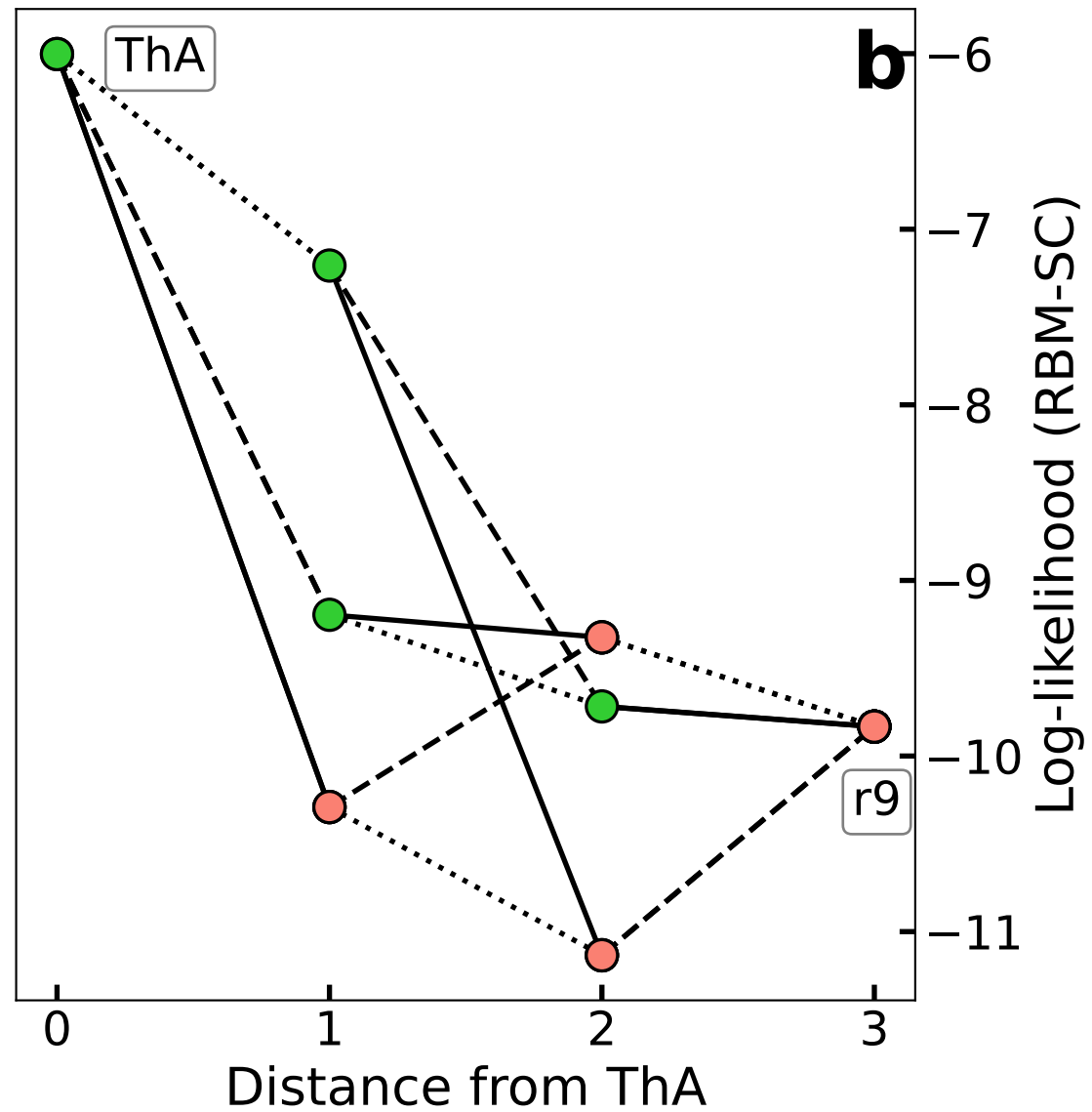

Supplement: S11 Fig — Different mutations are represented with different line styles: dotted lines for mutations involving position 5 (mutating A into T when going from ThA to r9), dashed lines for mutations involving position 8 (mutating A into G when going from ThA to r9), and solid lines for mutations involving position 17 (mutating A into T when going from ThA to r9). (PDF) [file pcbi.1010561.s011.pdf]

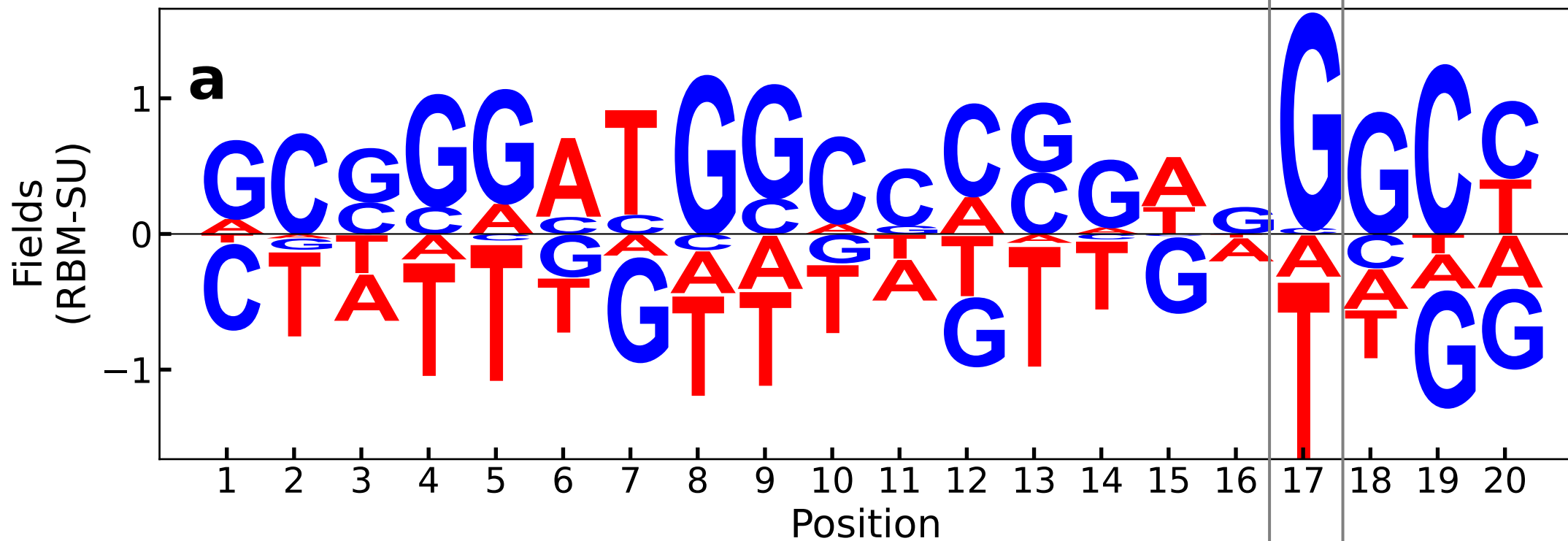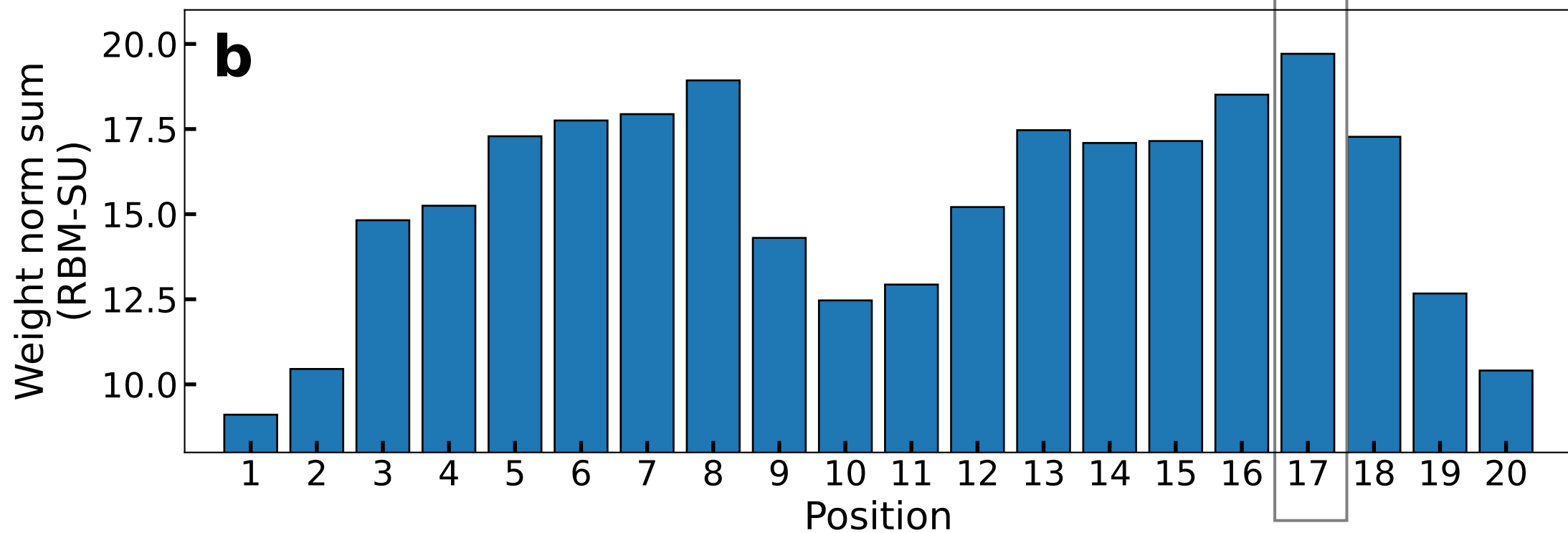

Supplement: S12 Fig — Panel A: fields of RBM-SU. The largest field (in norm) corresponds to position 17 (gray box), which is the one that in S11 Fig determines the binding exosite. Panel B: sum of the norms of each weight of RBM-SU, at fixed sequence position. The largest sum corresponds again to position 17 (gray box). (PDF) [file pcbi.1010561.s012.pdf]

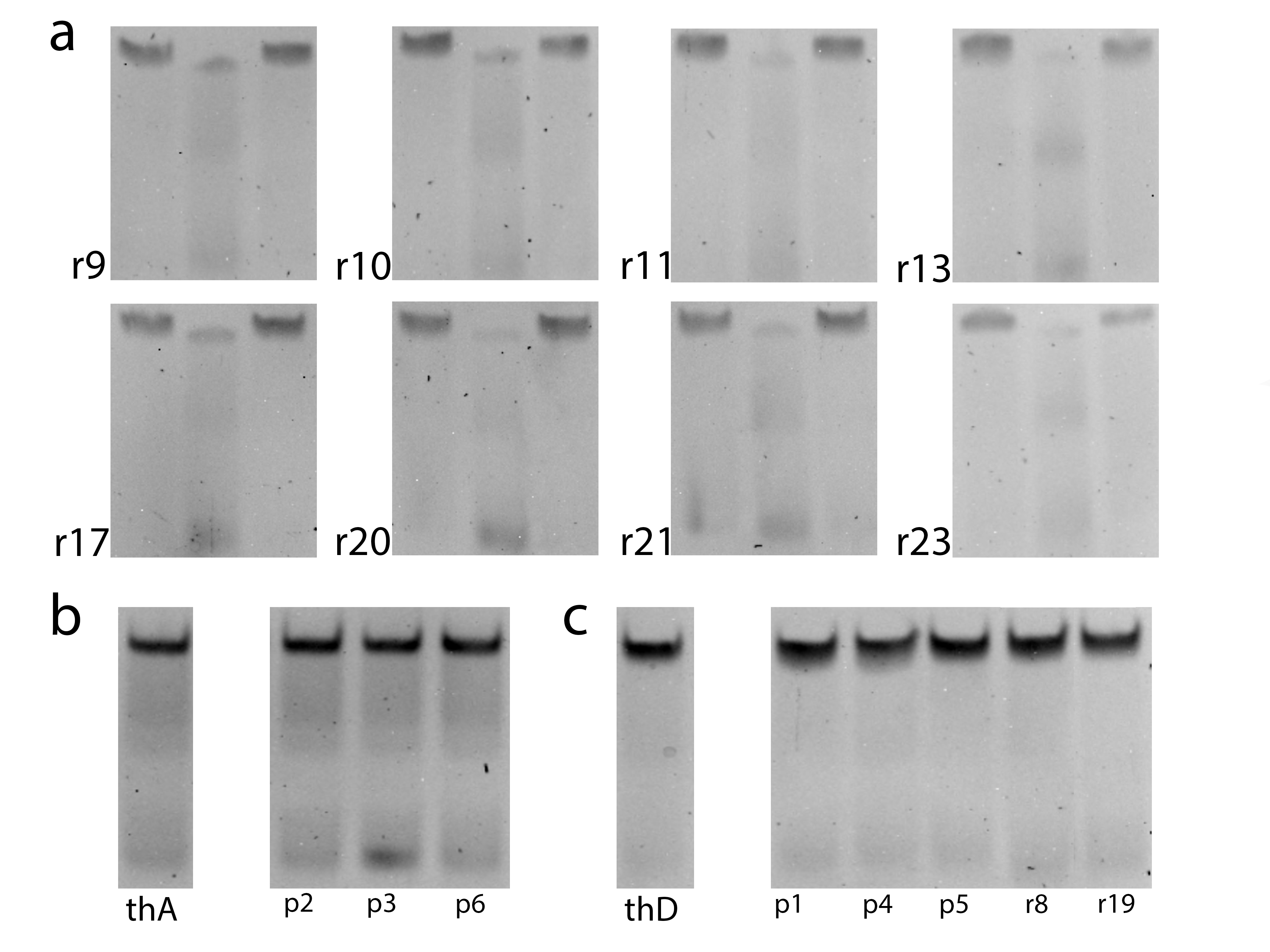

Supplement: S13 Fig — One sided competition assays of all exosite-I binders vs. a different fluorophore labeled strand in each well, r8, r14, and r19 respectively (panel A). Numbers to the left of each trial indicate the identity of the non-labeled strand. Additionally exosite-II binders were tested against fluorophore labeled ThA with negative control labeled ThA (panel B) and select exosite-I binders were tested against ThD with negative control labeled ThD (panel C). (PNG) [file pcbi.1010561.s013.png]

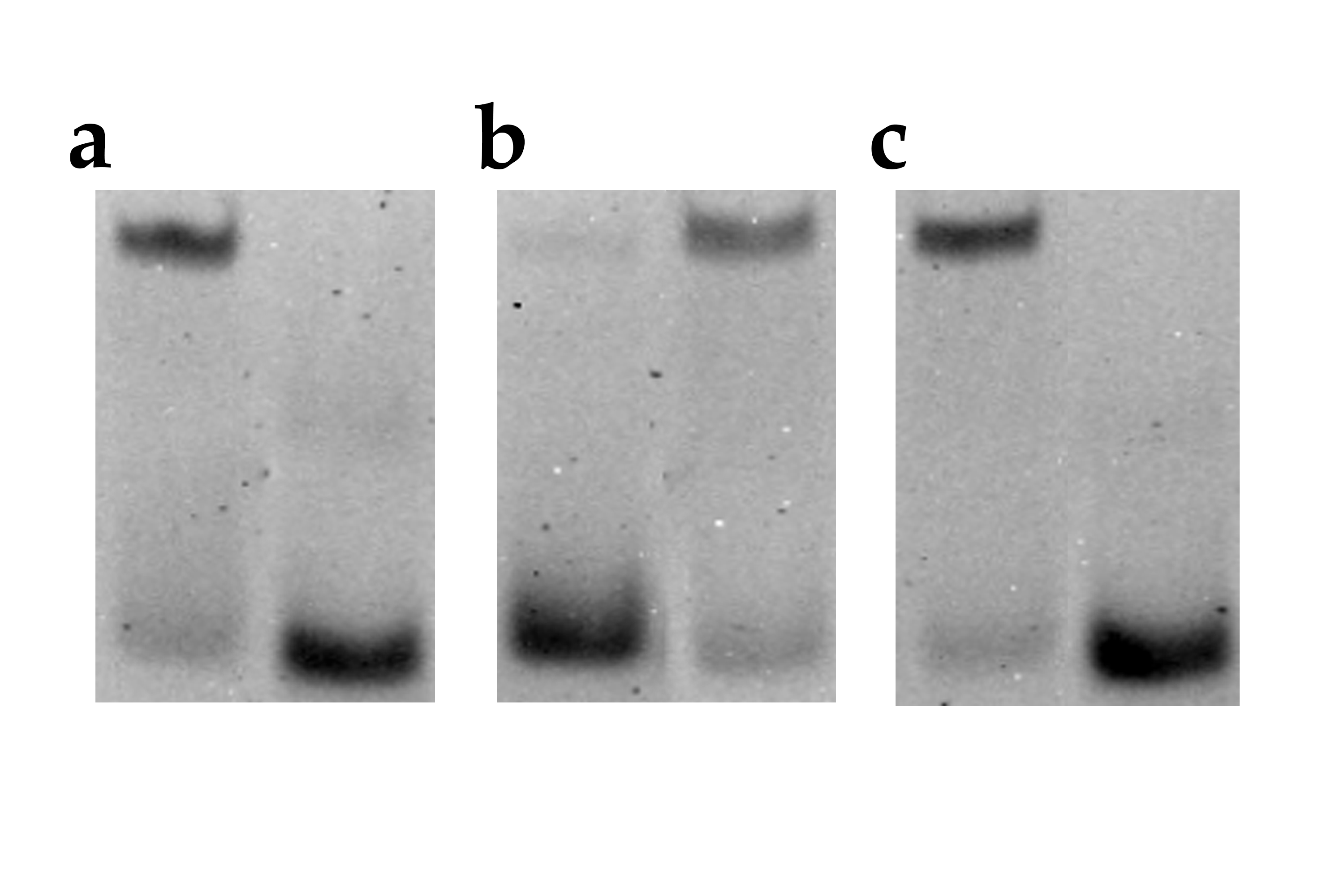

Supplement: S14 Fig — Competition assay of r8F vs r14 and r14F vs r8 (panel A). r8F vs r19 and r19F vs r8 (panel B), and r19F vs r14 and r14F vs r19 (panel C). The F suffix indicates the strand is fluorophore labeled with a 5’ 6FAM modification. (PNG) [file pcbi.1010561.s014.png]

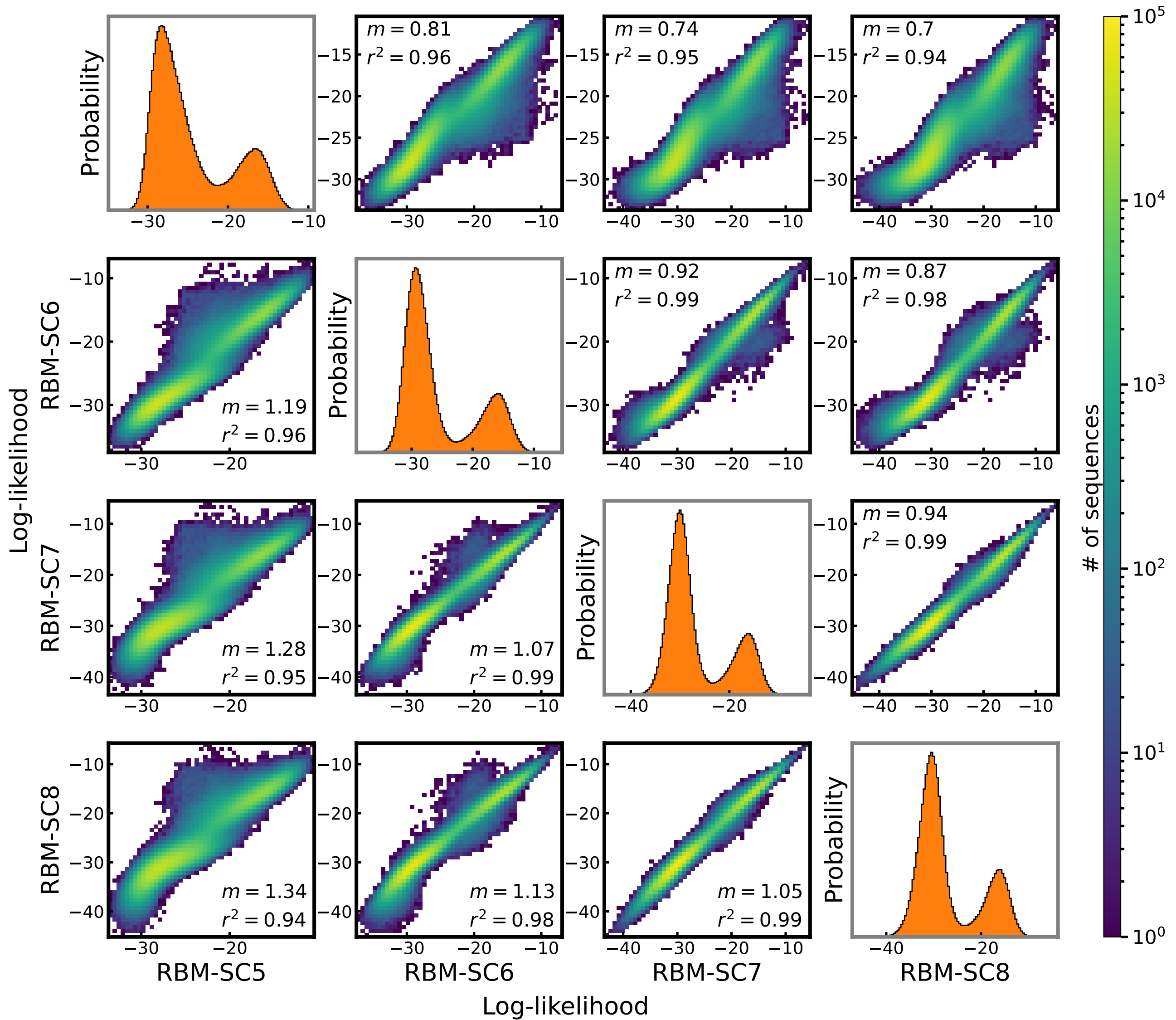

Supplement: S15 Fig — Plots on the diagonal are the distribution of the log-likelihoods of each RBM. The sequences used to prepare each histogram are the full set of sequences observed in round 5, 6, 7, or 8 (discarding counts). In each-non diagonal plot, the slope m and the coefficient of determination r2 for the linear fit are given. (PDF) [file pcbi.1010561.s015.pdf]

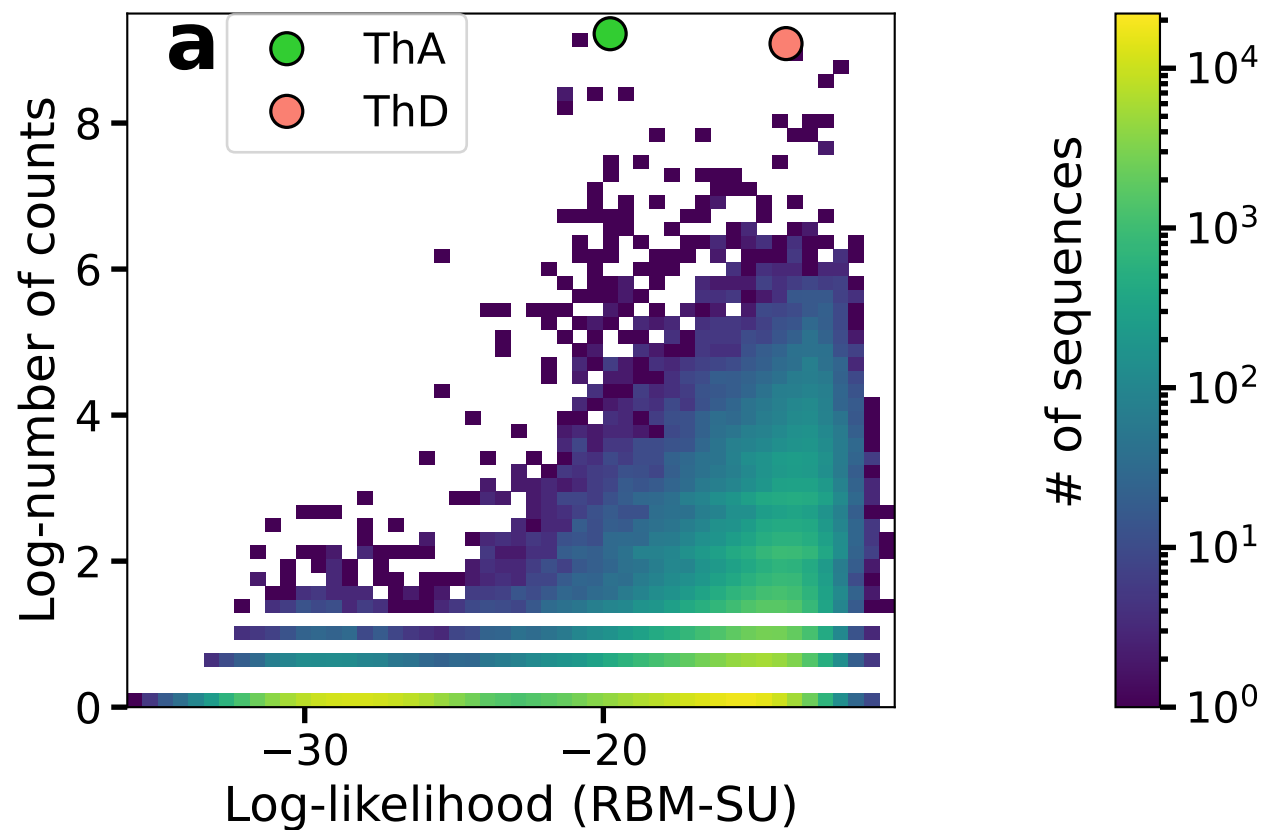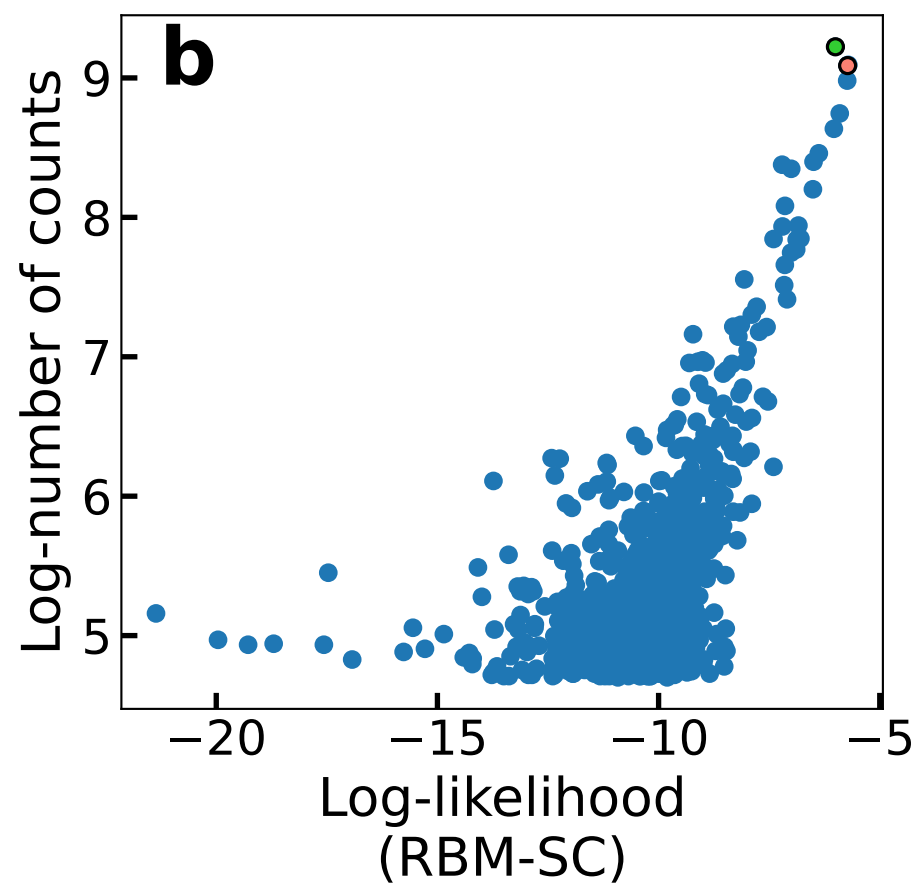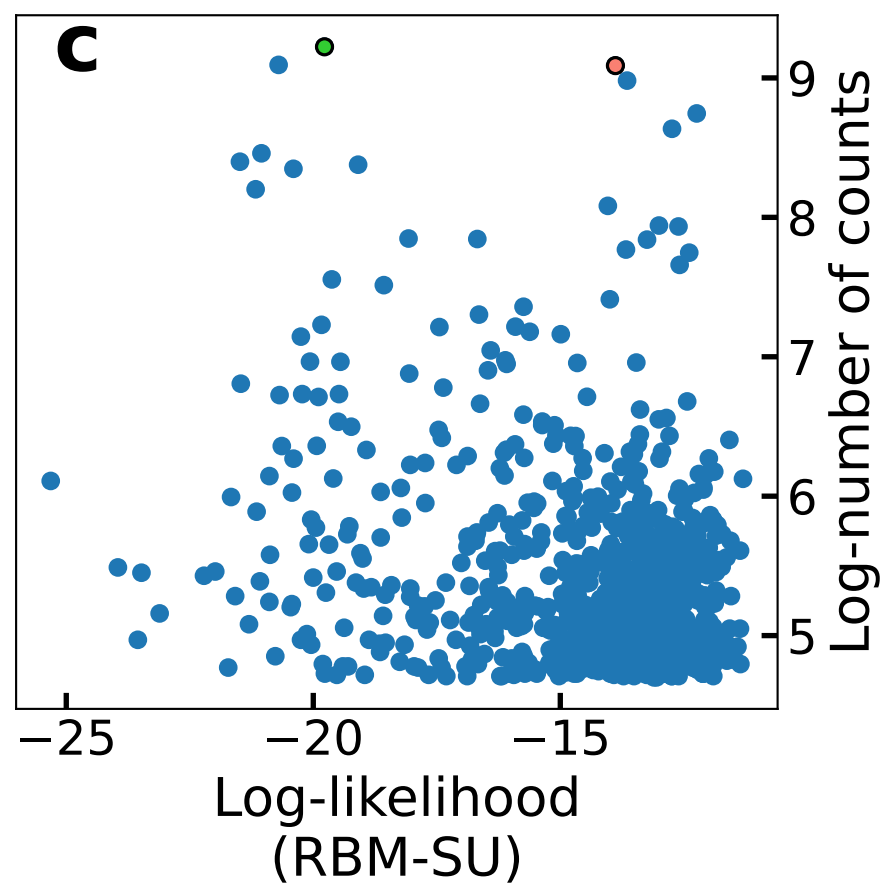

Supplement: S16 Fig — Panel A: Log-likelihoods (computed with RBM-SU) versus log number of counts for the unique single-loop sequences observed at round 8. ThA (counts: 10132, log-likelihood: -19.8) and ThD (counts: 8853, log-likelihood: -13.9) are highlighted with circles. Panels B, C: Log-likelihoods computed with RBM-SC (for panel B) or RBM-SU (for panel C) versus log number of counts for the 1000 unique single-loop sequences observed at round 8 with highest number of counts. (PDF) [file pcbi.1010561.s016.pdf]

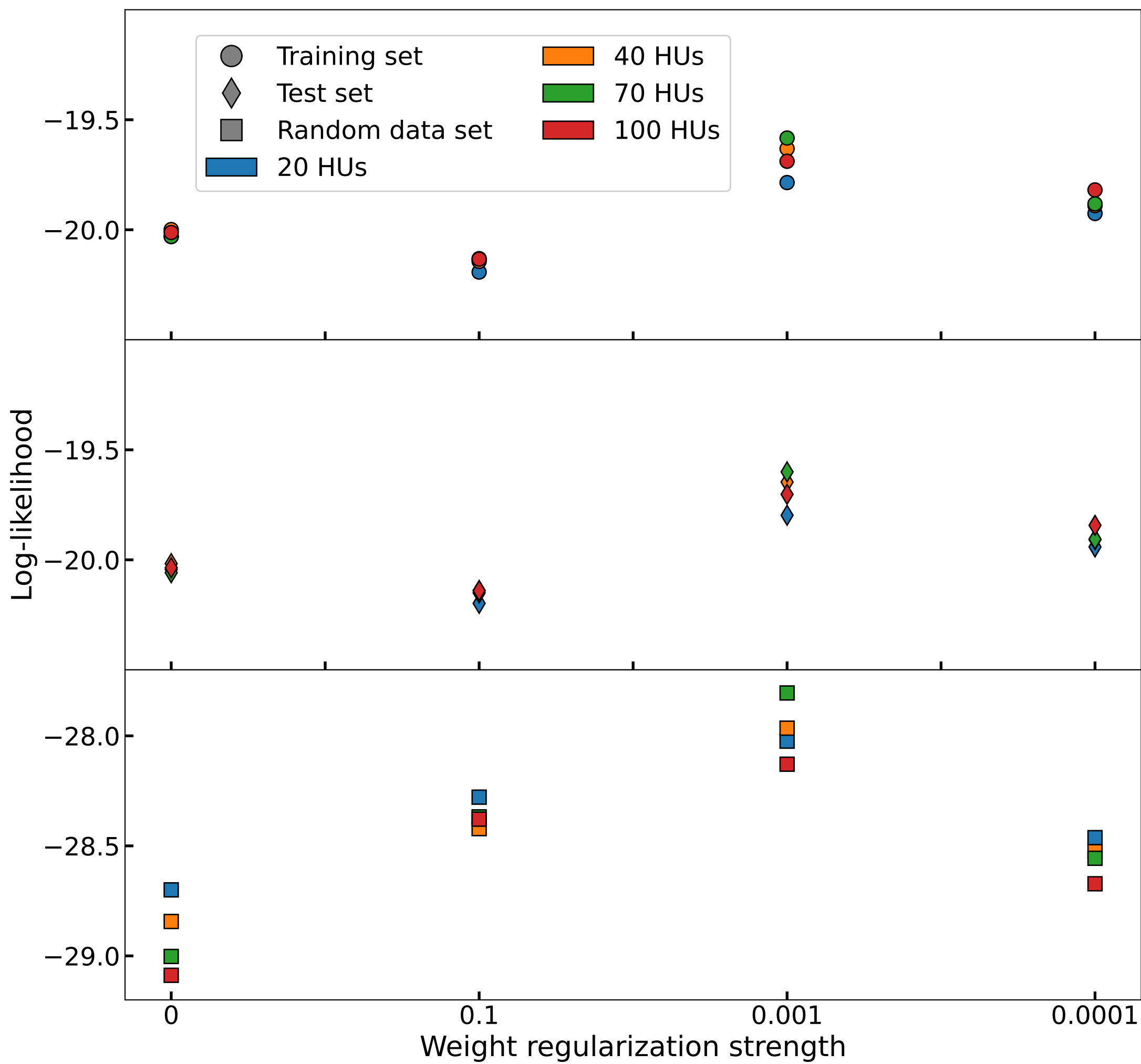

Supplement: S17 Fig — The scale on the y-axis is kept constant across the different sub-plots to highlight how the difference in average log-likelihoods are much smaller than the difference between the log-likelihood of training (and test) data and that of random sequences. The green circle at 0.001 regularization strength correspond to the RBM used in the paper (RBM-SU). (PDF) [file pcbi.1010561.s017.pdf]

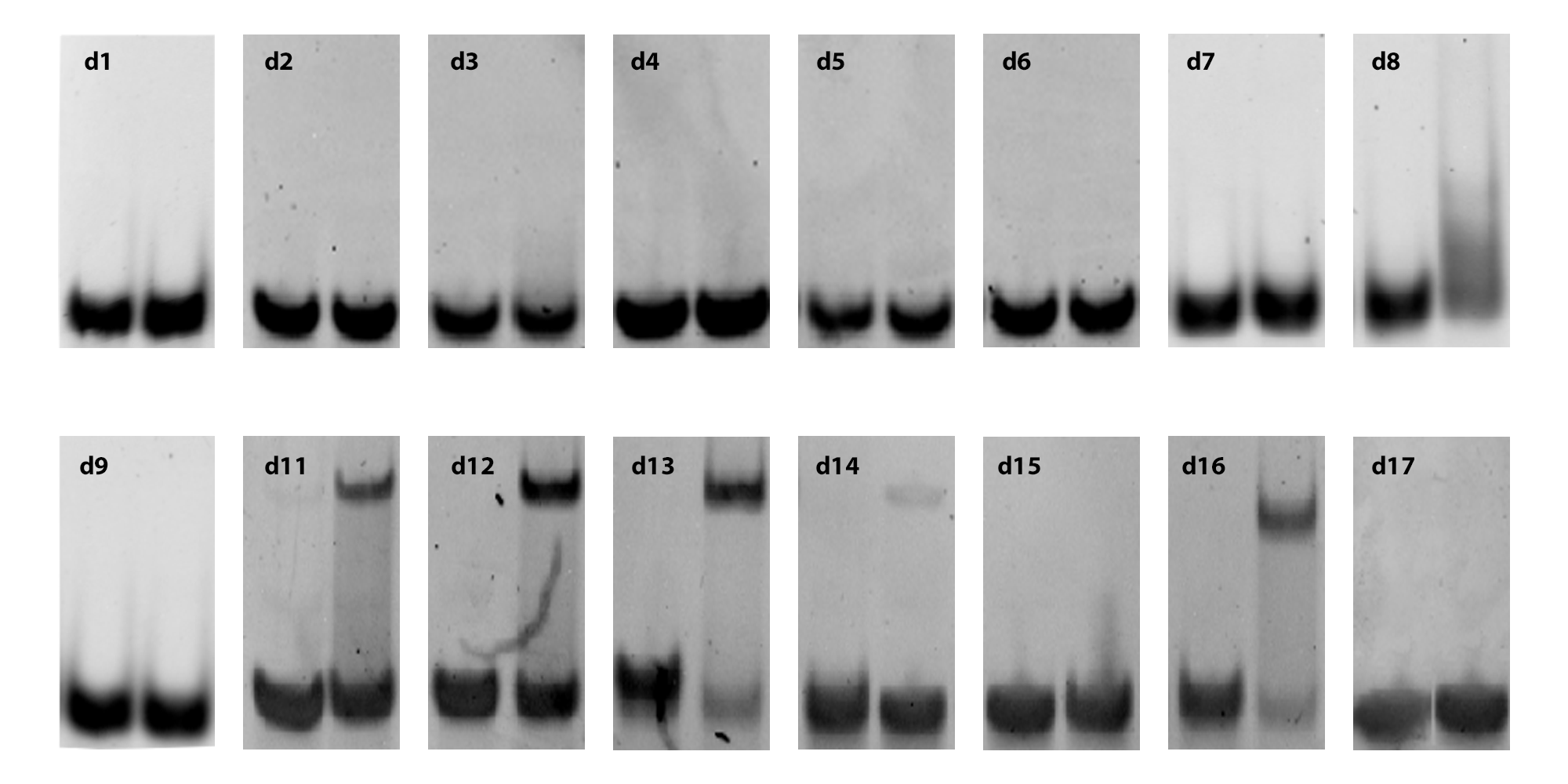

Supplement: S18 Fig — Lane 1 has the stem loop alone, whereas lane 2 has the same stem loop exposed to thrombin. Binding sequences are indicated by a high visible band in lane 2. (PNG) [file pcbi.1010561.s018.png]
