# Supplementary material for: Generative and interpretable machine learning for aptamer design and analysis of in vitro sequence selection
Source: PLoS Comput Biol. 2022 Sep 29;18(9):e1010561. doi: 10.1371/journal.pcbi.1010561 (PMC9553063; doi:10.1371/journal.pcbi.1010561)
Supplement: S1 Table — B indicates a binder while NB indicates a nonbinder. (PDF) [file pcbi.1010561.s019.pdf]

Label	Sequence	Score	Binder Prediction	Experimental result
d1	AGGGTAGGTGTGGGGTATGC	86.92	B	NB
d2	AGGGTAGATGTGTAGGATGC	87.86	B	NB
d3	AGGGATGATGGTTGGTAGGC	84.76	B	NB
d4	AGGGATGATGTGGATTAGGC	86.03	B	NB
d5	AGGGTGGGAGCGGGGGACGC	75.01	B	NB
d6	CGGGTAGGTGTGGATTATGC	77.59	B	NB
d7	GTAGGACGGGTAGGGCGGTC	67.57	NB	NB
d8	GGGGGTTGGGCGGGATGGGC	72.15	B	NB
d9	GCGGGTTGGGCAGGATCAGC	44.58	NB	NB
d10	AGGGATGATGTGTGGTAGGC	N/A	Cntrl	Cntrl
d11	GTAGGATGGGTGGGGTGGGA	86.46	B	B
d12	GTAGGATGGGTAGGGTGGTA	84.76	B	B
d13	CTAGGTTGGGTAGGGTGGTG	75.01	B	B
d14	CTAGCATGGGTAGGGTGGTG	77.59	B	B
d15	GTAGCATGGGTAGGGTGGTC	65.57	NB	NB
d16	TTGGGTGGTGTAGGTTGGCG	72.15	B	B
d17	TTGGGTGGTGCAGGTTCGCG	44.58	NB	NB
d18	CTAGGATGGGTAGGGTGGTG	N/A	Cntrl	Cntrl
