# Supplementary material for: Generative and interpretable machine learning for aptamer design and analysis of in vitro sequence selection
Source: PLoS Comput Biol. 2022 Sep 29;18(9):e1010561. doi: 10.1371/journal.pcbi.1010561 (PMC9553063; doi:10.1371/journal.pcbi.1010561)
Supplement: S2 Table — Here we fixed the fitness scale so that β6 = 1, or, equivalently, each value in this table is given in units of β6. Coefficients αr (with r = 5, 6, 7) can be obtained: (i) as the slopes in Fig 2C (first column); (ii) using the Fisher’s ratio, see inset of Fig 2C (second column); (iii) from the slopes in S15 Fig, since βr+1 − βr = αr, see Eq (3). Each of these methods has different noise sources, but the values obtained are in quite good agreement. (PDF) [file pcbi.1010561.s020.pdf]

	Fig 2C (slope)	Fig 2C (Fisher's ratio)	S15 Fig
β_5	-	-	0.81
β_6	-	-	1
β_7	-	-	1.07
β_8	-	-	1.13
α_5	0.16	0.14	0.19
α_6	0.07	0.06	0.07
α_7	0.01	0.01	0.05
