# Supplementary material for: Generative and interpretable machine learning for aptamer design and analysis of in vitro sequence selection
Source: PLoS Comput Biol. 2022 Sep 29;18(9):e1010561. doi: 10.1371/journal.pcbi.1010561 (PMC9553063; doi:10.1371/journal.pcbi.1010561)
Supplement: S3 Table — Since a good binder is expected to be found close to a sequence with many counts, we also provide in the other columns (Dist3, Dist10, Dist100) the distance to the closest single-loop aptamer with at least, respectively, 3, 10 or 100 counts in round 8 (respectively 74785, 22332, and 1177 sequences). (PDF) [file pcbi.1010561.s021.pdf]

Label	counts round 8	Dist1	Dist3	Dist10	Dist100
r1	3	0	0	1	1
r2	3	0	0	1	1
r3	0	1	1	1	1
r4	0	1	1	2	2
r5	0	1	1	2	2
r6	242	0	0	0	0
r7	341	0	0	0	0
r8	11	0	0	0	1
r9	9	0	0	1	2
r10	0	1	2	2	3
r11	0	2	2	2	4
r12	0	1	2	3	3
r13	0	2	2	3	5
r14	0	2	2	2	5
r15	0	2	2	2	4
r16	0	1	2	2	3
r17	0	1	2	3	4
r18	528	0	0	0	0
r19	139	0	0	0	0
r20	10	0	0	0	1
r21	8	0	0	1	2
r22	0	2	2	2	2
r23	0	1	1	2	4
r24	0	1	1	1	3
r25	0	1	1	2	3
r26	0	1	3	3	4
r27	0	1	1	1	3
