# Supplementary material for: Generative and interpretable machine learning for aptamer design and analysis of in vitro sequence selection
Source: PLoS Comput Biol. 2022 Sep 29;18(9):e1010561. doi: 10.1371/journal.pcbi.1010561 (PMC9553063; doi:10.1371/journal.pcbi.1010561)
Supplement: S4 Table — r1–27 correspond to sequences generated from sampling our RBM. All sequences with p labels (p1-p6) are along the mutation pathway from sequence ThA to r9. Sequences d1-d9 and d11-d17 are were generated from sampling from the DCA parameters. Sequences d10, d18, ThA, and ThD were used as controls throughout. (PDF) [file pcbi.1010561.s022.pdf]

| Sample Name | Full Reported Sequence |
|---|---|
| r1 | CTCGAGAGTTGCAGAAGTAGTGATGATGTGTGGTAGGCACTTCTGCAACTCTCGAG |
| r2 | CTCGAGAGTTGCAGAAGTAGTGTAGGTGTGGATGATGCACTTCTGCAACTCTCGAG |
| r3 | CTCGAGAGTTGCAGAAGTTAGGTTTTGGGTAGCGTGGTACTTCTGCAACTCTCGAG |
| r4 | CTCGAGAGTTGCAGAAGTAGGGATGATGTGTGGCAGGAACTTCTGCAACTCTCGAG |
| r5 | CTCGAGAGTTGCAGAAGTCTAGGACGGGTAGGGCGGTGACTTCTGCAACTCTCGAG |
| r6 | CTCGAGAGTTGCAGAAGTAGGGATGTGTGTGGTAGGCTACTTCTGCAACTCTCGAG |
| r7 | CTCGAGAGTTGCAGAAGTAGGGATGCTGCGTGGTAGGCACTTCTGCAACTCTCGAG |
| r8 | CTCGAGAGTTGCAGAAGTGAGGGTTGGTGTGGTTGGCAACTTCTGCAACTCTCGAG |
| r9 | CTCGAGAGTTGCAGAAGTAGGGTTGGTGTGTGGTTGGCACTTCTGCAACTCTCGAG |
| r10 | CTCGAGAGTTGCAGAAGTATGGTTGGTTTATGGTTGGCACTTCTGCAACTCTCGAG |
| r11 | CTCGAGAGTTGCAGAAGTGAAGGGTGGTCAGGGTGGGAACTTCTGCAACTCTCGAG |
| r12 | CTCGAGAGTTGCAGAAGTGGAGGGTGGGTCGGGTGGGAACTTCTGCAACTCTCGAG |
| r13 | CTCGAGAGTTGCAGAAGTGGGGTTGGTACAGGGTTGGCACTTCTGCAACTCTCGAG |
| r14 | CTCGAGAGTTGCAGAAGTAGATGGGCAGGTTGGTGCGGACTTCTGCAACTCTCGAG |
| r15 | CTCGAGAGTTGCAGAAGTAGATGGGTGGGTAGGGTGGGACTTCTGCAACTCTCGAG |
| r16 | CTCGAGAGTTGCAGAAGTATAGGGTGGGTGGGTGGGTAACTTCTGCAACTCTCGAG |
| r17 | CTCGAGAGTTGCAGAAGTTGGTGGTTGGGTTGGGTTGGACTTCTGCAACTCTCGAG |
| r18 | CTCGAGAGTTGCAGAAGTTGGGATGGGATTGGTAGGCGACTTCTGCAACTCTCGAG |
| r19 | CTCGAGAGTTGCAGAAGTAGGGTTGGTTATGTGGTTGGACTTCTGCAACTCTCGAG |
| r20 | CTCGAGAGTTGCAGAAGTATTGGTTGGGTAGGGTGGTTACTTCTGCAACTCTCGAG |
| r21 | CTCGAGAGTTGCAGAAGTAAACGGTTGGTGAGGTTGGTACTTCTGCAACTCTCGAG |
| r22 | CTCGAGAGTTGCAGAAGTCGGGGTGGTGTGGGTGGGAGACTTCTGCAACTCTCGAG |
| r23 | CTCGAGAGTTGCAGAAGTTATTGGTTGGATAGGTTGGTACTTCTGCAACTCTCGAG |
| r24 | CTCGAGAGTTGCAGAAGTAGGGTTGGGTGGTTGGATGAACTTCTGCAACTCTCGAG |
| r25 | CTCGAGAGTTGCAGAAGTCGGGTTGGGGGGTTGGATTCACTTCTGCAACTCTCGAG |
| r26 | CTCGAGAGTTGCAGAAGTCGGTTGGGGGGGTTGGATACACTTCTGCAACTCTCGAG |
| r27 | CTCGAGAGTTGCAGAAGTTGTGGGTTGGTGAGGTAGGTACTTCTGCAACTCTCGAG |
| ThA | CTCGAGAGTTGCAGAAGTAGGGATGATGTGTGGTAGGCACTTCTGCAACTCTCGAG |
| ThD | CTCGAGAGTTGCAGAAGTGTAGGATGGGTAGGGTGGTCACTTCTGCAACTCTCGAG |
| p1 | CTCGAGAGTTGCAGAAGTAGGGATGATGTGTGGTTGGCACTTCTGCAACTCTCGAG |
| p2 | CTCGAGAGTTGCAGAAGTAGGGATGGTGTGTGGTAGGCACTTCTGCAACTCTCGAG |
| p3 | CTCGAGAGTTGCAGAAGTAGGGTTGATGTGTGGTAGGCACTTCTGCAACTCTCGAG |
| p4 | CTCGAGAGTTGCAGAAGTAGGGATGGTGTGTGGTTGGCACTTCTGCAACTCTCGAG |
| p5 | CTCGAGAGTTGCAGAAGTAGGGTTGATGTGTGGTTGGCACTTCTGCAACTCTCGAG |
| p6 | CTCGAGAGTTGCAGAAGTAGGGTTGGTGTGTGGTAGGCACTTCTGCAACTCTCGAG |
| d1 | TCAGGCTCTCGAGAGTTGCAGAAGTAGGGTAGGTGTGGGGTATGCACTTCTGCCTGCATCGAGACA |
| d2 | TCAGGCTCTCGAGAGTTGCAGAAGTAGGGTAGATGTGTAGGATGCACTTCTGCCTGCATCGAGACA |
| d3 | TCAGGCTCTCGAGAGTTGCAGAAGTAGGGATGATGGTTGGTAGGCACTTCTGCCTGCATCGAGACA |
| d4 | TCAGGCTCTCGAGAGTTGCAGAAGTAGGGATGATGTGGATTAGGCACTTCTGCCTGCATCGAGACA |
| d5 | TCAGGCTCTCGAGAGTTGCAGAAGTAGGGTGGGAGCGGGGGACGCACTTCTGCCTGCATCGAGACA |
| d6 | TCAGGCTCTCGAGAGTTGCAGAAGTCGGGTAGGTGTGGATTATGCACTTCTGCCTGCATCGAGACA |
| d7 | TCAGGCTCTCGAGAGTTGCAGAAGTGTAGGACGGGTAGGGCGGTCACTTCTGCCTGCATCGAGACA |
| d8 | TCAGGCTCTCGAGAGTTGCAGAAGTGGGGGTTGGGCGGGATGGGCACTTCTGCCTGCATCGAGACA |
| d9 | TCAGGCTCTCGAGAGTTGCAGAAGTGCGGGTTGGGCAGGATCAGCACTTCTGCCTGCATCGAGACA |
| d10 | TCAGGCTCTCGAGAGTTGCAG AAGTAGGGATGATGTGTGGTAGGCACTTCTGCCTGCATCGAGACA |
| d11 | /5PHOS/CCAGTTTTTCTGGTGAGCTAGTGCAGACATGATCGTAGGATGGGTGGGGTGGGAGATCATGTAACTCCTAGCTGCCTGA |
| d12 | /5PHOS/CCAGTTTTTCTGGTGAGCTAGTGCAGACATGATCGTAGGATGGGTAGGGTGGTAGATCATGTAACTCCTAGCTGCCTGA |
| d13 | /5PHOS/CCAGTTTTTCTGGTGAGCTAGTGCAGACATGATCCTAGGTTGGGTAGGGTGGTGGATCATGTAACTCCTAGCTGCCTGA |
| d14 | /5PHOS/CCAGTTTTTCTGGTGAGCTAGTGCAGACATGATCCTAGCATGGGTAGGGTGGTGGATCATGTAACTCCTAGCTGCCTGA |
| d15 | /5PHOS/CCAGTTTTTCTGGTGAGCTAGTGCAGACATGATCGTAGCATGGGTAGGGTGGTCGATCATGTAACTCCTAGCTGCCTGA |
| d16 | /5PHOS/CCAGTTTTTCTGGTGAGCTAGTGCAGACATGATCTTGGGTGGTGTAGGTTGGCGGATCATGTAACTCCTAGCTGCCTGA |
| d17 | /5PHOS/CCAGTTTTTCTGGTGAGCTAGTGCAGACATGATCTTGGGTGGTGCAGGTTCGCGGATCATGTAACTCCTAGCTGCCTGA |
| d18 | /5PHOS/CCAGTTTTTCTGGTGAGCTAGTGCAGACATGATCCTAGGATGGGTAGGGTGGTGGATCATGTAACTCCTAGCTGCCTGA |
