# Supplementary material for: Generative and interpretable machine learning for aptamer design and analysis of in vitro sequence selection
Source: PLoS Comput Biol. 2022 Sep 29;18(9):e1010561. doi: 10.1371/journal.pcbi.1010561 (PMC9553063; doi:10.1371/journal.pcbi.1010561)
Supplement: S1 Appendix — (PDF) [file pcbi.1010561.s023.pdf]

Details of RBMs' training

We trained several RBMs which have been used for the analysis presented in this manuscript. For the training of each RBM, we used 90% of the dataset as training set and 10% of the dataset as validation set to check that no overfitting is observed. For RBMs trained using information on counts, the training dataset is obtained by sampling from the dataset of unique sequences many sequences (below the exact number for each RBM is given), with a probability proportional to each sequence's count (this gives the same results as long as the size of the re-sampled dataset is large enough, and allows to avoid having too large dataset which considerably slow down the RBM training). The training set was divided in mini-batches and for each epoch each mini-batch was used to perform an update of the parameters, using the persistent contrastive divergence algorithm with few (below the exact numbers are given) number of Monte-Carlo steps for each update of the paramters. In all cases the training stopped after 20000 updates of the RBM parameters. Finally, we used a L_1^2 regularization of the form given in Eq (6) to increase sparsity in the weights, which in turn improves the interpretability of the contribution of each hidden unit to a sequence's log-likelihood. Below we give the regularization parameter λ used for each RBM trained (see Eq (6)).

For the full range of explored hyperparameters (size of mini-batches, number of Monte-Carlo steps, regularization strength), we never saw any sign of relevant overfitting, and we motivated this with the very large datasets that are available for training the models.

The code used to train the RBMs can be obtained from github.com/jertubiana/PGM.

We now give more details about the training of each RBM model used in this manuscript. To distinguish RBMs trained with sequences observed in different rounds, we will append the round number to the model name. In particular, we used the following RBMs in this manuscript:

- RBM-DC6 (Fig 2, S1 Fig, S4 Fig), trained on the double aptamers (40 nucleotides) obtained from the SELEX 6th round. The training set is built by re-sampling 736436 sequences from the dataset of unique double-loop sequences observed in round 6, using their number of counts as weight for the sampling. The parameters are: 40 visible units, 90 hidden units, $\lambda = 0.01$, 10 Monte-Carlo steps for each update of the parameters, mini-batches of size 500.

- RBM-DC8 (Figs 3, 4, S7 Fig, S6 Fig), trained on the double aptamers (40 nucleotides) obtained from the SELEX 8th round. The training set is built by re-sampling 719413 sequences from the dataset of unique double-loop sequences observed in round 8, using their number of counts as weight for the sampling. The parameters are: 40 visible units, 90 hidden units, $\lambda = 0.01$, 10 Monte-Carlo steps for each update of the parameters, mini-batches of size 500.

- RBM-SC8 (Figs 3, 4, 8, Table 1, S3 Fig, S5 Fig, S16 Fig, S11 Fig, 8), trained on the single aptamers (20 nucleotides) obtained from the SELEX 8th round. The training set is built by re-sampling 725431 sequences from the dataset of unique single-loop left or right sequences observed in round 8, using their number of counts as weight for the sampling. The parameters are: 20 visible units, 80 hidden units, $\lambda = 0.01$, 2 monte-carlo steps for each update of the parameters, mini-batches of size 1000.

- RBM-SU8 (Figs 5, 8, Table 1, S6 Fig, S12 Fig, S16 Fig, S11 Fig, Fig 3 in S5 Appendix), trained on the single aptamers (20 nucleotides) obtained from the SELEX 8-th round, merging sequences from the left and right loops (unique single-loop sequences: 382094; with counts: 1450862). Multiple copies of the same aptamer are neglected. The parameters are: 20 visible units, 70 hidden units, $\lambda = 0.01$, 4 Monte-Carlo steps for each update of the parameters, mini-batches of size 500.

- RBM-SC5 (S15 Fig), trained on the single aptamers (20 nucleotides) obtained from the SELEX 5th round. The training set is built by re-sampling 1375403 sequences from the dataset of unique single-loop left or right sequences observed in round 5, using their number of counts as weight for the sampling. The parameters are: 20 visible units, 70 hidden units, $\lambda = 0.01$, 8 monte-carlo steps for each update of the parameters, mini-batches of size 1500.

- RBM-SC6 (S15 Fig), trained on the single aptamers (20 nucleotides) obtained from the SELEX 6th round. The training set is built by re-sampling 598696 sequences from the dataset of unique single-loop left or right sequences observed in round 6, using their number of counts as weight for the sampling. The parameters are: 20 visible units, 80 hidden units, $\lambda = 0.01$, 8 monte-carlo steps for each update of the parameters, mini-batches of size 600.

- RBM-SC7 (S15 Fig), trained on the single aptamers (20 nucleotides) obtained from the SELEX 7th round. The training set is built by re-sampling 419934 sequences from the dataset of unique single-loop left or right sequences observed in round 7, using their number of counts as weight for the sampling. The parameters are: 20 visible units, 70 hidden units, $\lambda = 0.01$, 8 monte-carlo steps for each update of the parameters, mini-batches of size 500.

- RBM-SU6 (S4 Fig), trained on the single aptamers (20 nucleotides) obtained from the SELEX 6-th round, merging sequences from the left and right loops (unique single-loop sequences: 598696; with counts: 1472872). Multiple copies of the same aptamer are neglected. The parameters are: 20 visible units, 70 hidden units, $\lambda = 0.01$, 4 Monte-Carlo steps for each update of the parameters, mini-batches of size 600.

- RBM-LC8, RBM-RC8 (S3 Fig), trained on the single aptamers (20 nucleotides) obtained from the SELEX 8-th round. The training sets of RBM-LC8 (RBM-RC8) is built by re-sampling 177014 (227789) sequences from the dataset of unique left-loop (right-loop) sequences observed in round 8, using their number of counts as weight for the sampling. The parameters of both models are: 20 visible units, 70 hidden units, $\lambda = 0.01$, 4 Monte-Carlo steps for each update of the parameters, mini-batches of size 500.

- RBM-NPU8 (S5 Fig), trained on the single aptamers (20 nucleotides) obtained from the SELEX 8-th round, merging sequences from the left and right loops, after excluding parasite sequences. Parasite sequences are obtained here as single-loop sequences with log-likelihood computed by RBM-SU8 lower than -24.8, with the partner loop having log-likelihood computed by RBM-SU8 larger than -24.8 (procedure resulting in 276682 unique single-loop non-parasite sequences). Multiple copies of the same aptamer are neglected. The parameters are: 20 visible units, 70 hidden units, $\lambda = 0.01$, 4 Monte-Carlo steps for each update of the parameters, mini-batches of size 500.

- RBM-NPC8 (S5 Fig), trained on the single aptamers (20 nucleotides) obtained from the SELEX 8-th round, merging sequences from the left and right loops, after excluding parasite sequences. Parasite sequences are obtained here as single-loop sequences with log-likelihood computed by RBM-SC8 lower than -26.6, with the partner loop having log-likelihood computed by RBM-SC8 larger than -26.6 (procedure resulting in 274250 unique single-loop non-parasite sequences). The training dataset is built by sampling 246825 non-parasite sequences, using their number of counts as weight for the sampling. The parameters are: 20 visible units, 70 hidden units, $\lambda = 0.01$, 4 Monte-Carlo steps for each update of the parameters, mini-batches of size 500.

All the parameters for the training which are not given here are the default parameters as defined in the code. The trained RBMs are provided in the Github repository (github.com/adigioacchino/RBMsForAptamers), together with a jupyter notebook that can be used to re-train them.

As a final remark, we checked that the results obtained here depend very little on the precise values of the hyperparameters used here (see S17 Fig). The only notable exception being the usage of counts to weight multiple occurrences of the same aptamer in the dataset. We decided to exclude multiple occurrences from the training to regularize the RBM, as discussed in in details in S9 Fig.
