# Supplementary material for: Generative and interpretable machine learning for aptamer design and analysis of in vitro sequence selection
Source: PLoS Comput Biol. 2022 Sep 29;18(9):e1010561. doi: 10.1371/journal.pcbi.1010561 (PMC9553063; doi:10.1371/journal.pcbi.1010561)
Supplement: S2 Appendix — (PDF) [file pcbi.1010561.s024.pdf]

Detailed experimental methods

Gel Electrophoresis

The formation of the Thrombin/DNA complex is sensitive to temperature as well as the presence of K^+. Therefore, each 5% native gel incorporated K^+ into its matrix and was run at 15C for 90 minutes at 200V. Running buffer was 10mM K^+ 7mM Mg^{2+} 1x TAE at 8 pH. Each gel was stained with SYBR gold prior to imaging at 300nm. If using a fluorophore labeled strand, the gel was was not stained prior to imaging.

DNA Sample Preparation

Each RBM-generated 20nt sequence was supplemented with two complementary 18nt regions to form a stem loop structure (56nt total). The RBM-generated stem loops were designed and their secondary structure predicted (see Fig 1 in S2 Appendix) using NUPACK's webserver [1]. NUPACK results showed no other complex formation except for the desired stem loop. The sequences were ordered, HPLC purified from IDT and re-suspended in 10mM K^+ 7mM Mg^{2+} 1x TAE. The stem loops were annealed for 12hrs to ensure proper secondary structure formation, and their concentrations standardized to 500nM by measure of the 260nm absorbance using a Nanodrop Spectrometer. All DCA-generated sequences were originally designed to form the nanotile from the SELEX experiment which generated our dataset [2]. Using each loop individually resulted in a 15nt stem loop with non-pairing regions. These sequences were ordered in a plate from IDT with their standard desalting. Each DCA-generated sequence was purified by using a 5% or 6% denaturing gel (depending on the sequence size) in 1x TBE buffer, cutting the resulting band and precipitating the DNA out with ethanol. The stem loops were annealed for 12hrs to ensure proper secondary structure formation, and their concentrations standardized to 500nM by measure of the 260nm absorbance using a Nanodrop Spectrometer. All sequences used throughout the main text are shown in S4 Table except for any 5' 6FAM modifications which are marked in any figures in which they are used.

Control Sequence Verification

To confirm the binding band and establish the interaction between the stem loop sequences and thrombin, the control strands ThA and ThD were exposed to varying concentrations of thrombin shown in S10 FigB and S10 FigC. For both ThA and ThD, the almost complete uptake of the stem-loop from the starting position (S10 Fig) to the stem-loop / complex band at a ratio of 1:1.08 indicates the stem loop / complex band interaction is made up of a single stem loop binding to a single molecule of thrombin. Further, the combination of the two stem loops binding to thrombin at the same concentrations (S10 Figd) confirmed the cooperative binding seen in previous experiments as well as indicated a downshift of the stem-loop / protein band upon 2 stem-loops binding to thrombin.

Competition Assays

Competition assays were performed by mixing equimolar amounts (2.5um) of a fluorophore labeled DNA strand and non-labeled DNA strand that bind to the same Thrombin exosite. The reverse is simultaneously tested, where the fluorophore labeled version of the non-labeled strand is substituted with a fluorophore labeled version and the previously non-labeled strand is substituted for a fluorophore labeled version. In both, Thrombin is added in a 1:2 ratio (2.5um) and allowed to mix at 25°C for 30 min. Comparing the results of the assays yields a conclusive ranking of the relative binding affinity of the two sequences. Competition assays using 5' 6FAM modified sequences are depicted in S14 Fig.

Additionally, one-sided competition assays for all exosite-I binders and all sequences between r9 and ThA were tested against fluorophore-labeled versions of the best binding aptamers from the previous study (ThA and ThD) to assess whether any novel binder performed better. From S13 FigB and S13 FigC we see that no exosite-II binding aptamer was found which bound better than ThA and no exosite-I binding aptamer was found which bound better than ThD. Additionally, the tested exosite-I binders S13 Fig(a) are worse binders than r8 and r19 but better binders than r14.

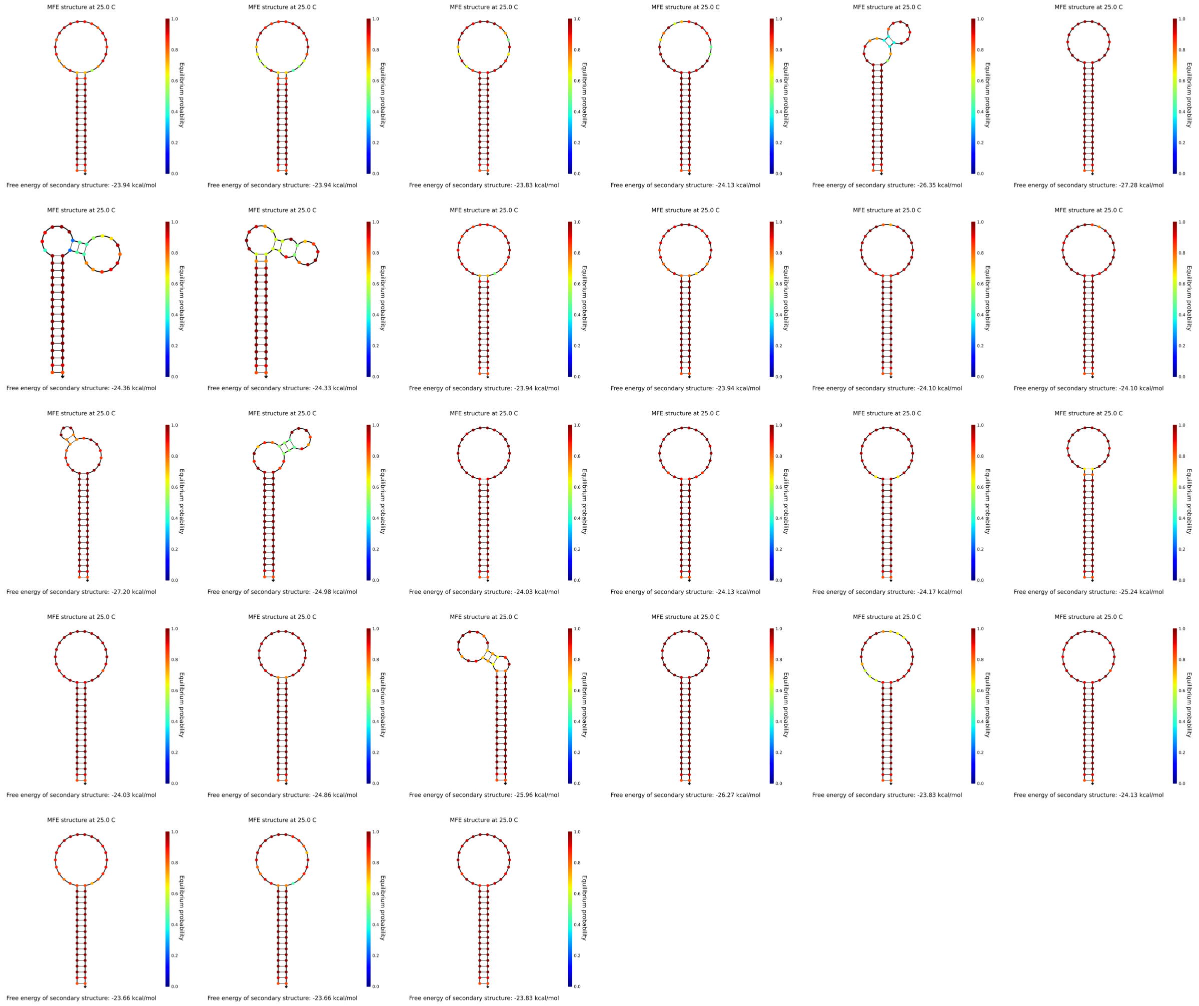

Figure 1: NUPACK Predictions of the minimum free energy structure (MFE) of each DNA stem-loop at 25 °C. Figures start from r1 in the top left corner to r27 in the bottom right corner.

Thrombin Sample Preparation

We used 1 mg Human α-thrombin manufactured by Haematologic Technologies Inc. and purchased from Fisher Scientific Co. Concentrations were assessed by 280nm absorbance using a Nanodrop Spectrometer. The stock was stored at -20C. Sample concentrations were made at 500nm and 250nm in 1x PBS 10mM K^{+} 7mM Mg^{2+}. Each sample was made fresh prior to being used in an assay.

References

1. Zadeh JN, Steenberg CD, Bois JS, Wolfe BR, Pierce MB, Khan AR, et al. NUPACK: Analysis and design of nucleic acid systems. Journal of Computational Chemistry. 2011;32(1):170–173. doi:10.1002/jcc.21596.

2. Zhou Y, Qi X, Liu Y, Zhang F, Yan H. DNA-Nanoscaffold-Assisted Selection of Femtomolar Bivalent Human alpha-Thrombin Aptamers with Potent Anticoagulant Activity. ChemBioChem. 2019;20(19):2494–2503. doi:https://doi.org/10.1002/cbic.201900265.
