# Supplementary material for: Generative and interpretable machine learning for aptamer design and analysis of in vitro sequence selection
Source: PLoS Comput Biol. 2022 Sep 29;18(9):e1010561. doi: 10.1371/journal.pcbi.1010561 (PMC9553063; doi:10.1371/journal.pcbi.1010561)
Supplement: S3 Appendix — (PDF) [file pcbi.1010561.s025.pdf]

Comparison with DNN and Traditional Machine Learning approaches

Dataset preparation

Starting with the raw SELEX data from the 8th round of selection of our previous study, we have both 20nt aptamer sequences in each arm of the DNA scaffold and a copy number, representing the number of times that sequence was observed during sequencing. Any sequence not matching the 40nt length was assumed to have a reading error and excluded from the dataset. Independent counts for each arm of the sequence were generated by counting their occurrence throughout the dataset. Using their individual counts, each 20nt sequence was categorized as either as a "good" (copy number > 10) or "bad" (copy number < 10) binder. Note that this approach is distinct from our training of the RBMs, where we considered all sequences in the training sample and used counts for weighting the sequences.

Our analysis of the dataset found a subset of bad binder sequences far in sequence space from any other observed sequence in the dataset that were paired with good binder sequences. We concluded that these sequences were most likely carried through the selection process by their good binder and subsequently excluded these sequences from our training set.

Three datasets were generated from the remaining sequences: sequences from the left loop (L), sequences from the right loop (R), and sequences from both loops (B). Each dataset consists of the entire set of good binders from the appropriate loop and 5 randomly sampled bad binders per good binder. Training sets (80% of good binders, $\sim 35k$ in total sequences for L and R, $\sim 70k$ for B) and validation sets (20% of good binders, $\sim 12k$ in total sequences for L and R, $\sim 25k$ for B) were split from our dataset. As further verification of our DNN and traditional ML models we used the experimental results from both the RBM-generated sequences as well as the DCA-model generated sequences to assess our models accuracy. All sequences were one-hot encoded prior to training, validation, or prediction.

As using only sequences from the final round of the SELEX procedure introduces a general bias of all sequences interacting with thrombin, three more datasets were created (GL, GR, and GB) with good binders selected as previously done but bad binders were randomly sampled from a set of random sequences outside the SELEX dataset's sequence space. These datasets had the same amount of sequences as those mentioned previously (L, R, B). We assume that if there is no bias in the initial random library, most of the possible aptamer sequences of length 20 were initially present, and hence a randomly generated sequence which is not encountered in the SELEX dataset is most likely not going to be able to bind to thrombin.

Model Selection

For the classification task we used 5 different deep learning models: 2 versions of a Variational Auto Encoder [1], 2 versions of a Resnet [2] and a Siamese Network Model [3] outlined in Table 1 in S3 Appendix. A schematic description of the DNN model specifications used in this work is provided in Table 1 in S3 Appendix. Additionally we used 3 classic Machine Learning methods: a decision tree, a random forest and a gradient boosted tree classifier to also classify the sequences as binders or non binders.

DNN Training Specifics

All 5 models were written as pytorch lightning modules and hyperparameter optimization was done using the raytune library. Integration of each pytorch module with raytune enabled simultaneous distributed hyperparameter optimization. All models were trained for either 30 or 50 epochs. No significant performance increase or decrease was observed between models trained for 30 vs 50 epochs.

Hyperparameter optimization was performed using the raytune library. For resnet, we optimized the batch size, learning rate (lr) and dropout (dr) prior to the dense layer and softmax. For variational Auto-Encoders, we optimized the batch size, learning rate, dropout and z_dim (embedding dimension). For the siamese network, we optimized the learning rate, batch size, and distance cutoff (Euclidean distance cutoff, being less means a match while being greater indicates a nonmatch). As a grid search, the AsyncHyperBandScheduler (AHSA) was given 10 trials with the goal to find the model with best accuracy on the validation set. Bayesian Optimization was performed on the same hyperparameters as the ASHA, save the integer valued batch size. Bayesian optimization was given a different directive,

**Model**	**Description**
Long Resnet	A 152 layer Resnet followed by a dropout layer, a 512 input to 2 output linear layer followed by a softmax layer. Residual networks guarantee performance of subsequent layers in the network by mapping to a residual function F(x) = H(x)-x. This network architecture has been shown to avoid vanishing gradients and accuracy degradation present in traditional network architecture learning [2]. During training, this model used label smoothed [4] (smoothing=0.01) cross entropy as its loss function.
Short Resnet	An 18 layer Resnet followed by a dropout layer, a 512 input to 512 output linear layer, a DReLU activation function, a 512 input to 2 output linear layer, and finally a softmax layer. During training, this model used label smoothed (smoothing=0.01) cross entropy as its loss function.
Long Variational AutoEncoder	A 2d convolution with ReLU activation function followed by three encoder blocks encoded the embedding. Encoder blocks consisted of a spectral normalized 2d convolution layer [5], followed by 2d batch normalization and a leaky ReLU activation function. The decoder consisted of 4 decoder blocks made up of a transposed 2d convolution followed by 2d batch normalization and a leaky ReLU activation function. Self attention layers were added in between both encoder and decoder blocks [6]. Binary classification of binder vs. nonbinder was performed on each embedding by two fully connected layers (sizes 128 and 64, consisting of: a dropout layer, linear layer, 1d batch norm, and a leaky ReLU) followed by a dropout layer, linear layer (size 2), and a final softmax layer. Similarly mu and logvar were generated by two fully connected layers and a final layer (sizes 128, 112, 100). Variational AutoEncoders are generative models designed to sample across a continuous latent space [1]. During training this model used label smoothed (smoothing=0.01) cross entropy on the predictions and symmetric MSE loss on the decoder's reconstruction. The loss functions were mixed for the total training loss.
Short Variational AutoEncoder	A 152 layer Resnet encoder and 2 decoder blocks (separated by an attention layer). A 512 input to 2 output linear layer was trained on each embedding with a log softmax layer on the end to predict a binding vs. nonbinding result. During training this model used label smoothed (smoothing=0.01) cross entropy on the predictions and symmetric MSE loss on the decoder's reconstruction. The loss functions were mixed for the total training loss.
Siamese	A Siamese network trained on pairs of sequences to discriminate between binder-binder pairs and nonbinder-binder pairs. The Siamese network used here consisted of a single resnet made up of 4 layers to a 512 input to 256 output linear layer, a sigmoid activation function, and a 256 input to 2 output linear layer following. Each iteration was run individually on pairs of sequences [3]. The Euclidean distance between the resulting embeddings is used to assign our binary classification value. During training this model used contrastive loss as its loss function.

Table 1: Descriptions of all DNN models used in this work.

to minimize the mean loss (training+validation). Population-based training was only performed on the siamese network with the goal of maximizing the accuracy on the validation set.

DNN Results

To compare performance of our DNN models, we assessed the accuracy of each model to predict a binder/nonbinder label for each experimentally validated dataset: the RBM generated dataset and the DCA generated dataset. We also calculated the F1 score metric by comparison of each model's prediction with the ground truth. The F1 score is the harmonic mean of precision, the number of true positives divided by the sum of true positives and false positives, and recall, the number of true positives divided by the sum of true positives and false negatives, in a binary classification task. A F1 score was calculated for each dataset and a mean F1 score was determined by weighting each individual F1 score by the number of total sequences in the dataset. Scores for the DNN models are provided in Table 2 in S3 Appendix.

DNN (L, R, B) models (i.e. models trained on L, R or B dataset) failed to generalize to our experimental datasets. In every case, prediction of binding ability on the RBM and DCA datasets results in a significant number of false positives and false negatives. Bayes hyperparameter optimized models were directed to either minimize the loss on the validation dataset or maximize the accuracy on the validation dataset whereas AsyncHyperBandScheduler (AHSA) hyperparmater optimized models were directed to only maximize the accuracy on the validation dataset. The most accurate (L, R, B) models on the RBM generated dataset (Bayes Resnet L and Bayes Resnet L and R) were directed to minimize the loss for hyperparameter optimization and achieved 74.1% (20/27) accuracy on the RBM experimental dataset with poor performance on the DCA generated dataset at 31.3% (5/16) accuracy. A distinct correlation between optimization directive and performance metrics was observed. Models that were optimized to minimize the loss of the validation dataset performed worse in validation set accuracy, better in RBM generated dataset binder prediction, and worse in DCA generated dataset binder prediction to a significant degree than those optimized to maximize the validation set accuracy. From the confusion matrices of loss minimized models on the RBM generated dataset (Fig 2 in S3 Appendix), we see these models are completely unable to distinguish between nonbinders and binders in both the RBM generated and DCA generated datasets.

DNN (L, R, B) models trained to maximize the accuracy on the validation set performed poorly overall. The best performing of them (ASHA VAE short R) managed the highest mean F1 score, excellent accuracy on the DCA generated dataset at 87.5% (14/16) accuracy but poor performance on the RBM generated dataset with 48.1% (13/27) accuracy. The poor performance of all DNN (L, R, B) models indicates the sequencing info of the last round of selection is not sufficient for DNN models to classify sequences on their ability to bind a target.

DNN (GL, GR, GB) models were trained as a more naive classifier using good binders and randomly generated bad binders for both training and validation. As the random bad binders were guaranteed be to outside the sequence space of the entire 8th round of selection, we would expect these models to over-predict binders in our datasets which contain binders and nonbinders separated by small distances in sequence space. Indeed, all (GL, GR, GB) models have higher accuracy values than their (L, R, B) counterparts, but consistently have little to none false negatives and a large number of false positives on the sequences generated using RBM (Fig 3 in S3 Appendix). Additionally the higher accuracy scores on the RBM generated dataset and lower accuracy scores on the DCA generated dataset is due to the difference in population group membership (binder vs. nonbinder) of the two datasets. Their ability to predict thrombin binding ability from sequences close in sequence space is subpar due to their overfitting to the aptamer sequence space.

The performance of all DNN models on predicting thrombin binding ability from sequence alone was poor. DNN (L, R, B) models tend to generate a notable amount of false positives and false negatives, while (GL, GR, GB) models generate false positives almost exclusively on the RBM generated dataset. Overall, using the last round of selection for our dataset exclusively (L, R, B) or for just the good binders (GL, GR, GB) did not allow accurate prediction of thrombin binding ability from any of the DNN models.

Traditional ML Training Specifics

Three traditional models: a single tree, a random forest, and a gradient-boosted forest were used to classify the experimental dataset as binders or nonbinders. The training and validation datasets used

were the same as those used for the deep learning models. The scikit-learn python library implementations of each of the three models were used in this work.

Traditional ML Results

Our traditional ML techniques' performance was measured by the same metrics as for our DNN models, namely the accuracy on the RBM generated sequences, the accuracy on the DCA generated sequences, and the F1 mean of both datasets shown in Table 3 in S3 Appendix.

Traditional (L, R, B) models very rarely predicted a nonbinder correctly in our RBM generated dataset, instead predicting almost every sequence to be a binder. Their validation set accuracy never crossed 30%. Similar to our DNN models, the accuracy on the validation sets of the (GL, GR, GB) models was significantly better than (L, R, B) models due to the difference in sequence space of the bad binders. Traditional (GL, GR, GB) models suffered from the same issue of an overabundance of false positives including the single tree models which had the best performance of any machine learning model besides the RBM. The GR single tree achieved an accuracy of 85.2% (23/27) on the RBM generated dataset and an accuracy of 81.3% on the DCA generated dataset. Despite the high accuracy, these models suffer from the same over-fitting that the DNN (GL, GR, GB) models where binders are over-predicted significantly. The small difference in single tree models GR and GB illustrate how decreasing the amount of false positives by one in the RBM generated set has the effect of predicting almost 20% less binders in the DCA generated dataset. This ability to overestimate binders is especially apparent in the confusion matrices of the random forest (GL, GR, GB) models Fig 4 in S3 Appendix. The random forest on average performed worse than the single tree, performing as well as most DNN models. This is in stark contrast to our gradient boosted classification tree which performed poorly on every dataset no matter the hyperparameters tried.

Additional ML Results

The main results for the DNN models and traditional ML models referenced in the main text are shown in Tables 2 and 3 in S3 Appendix respectively. Fig 1 in S3 Appendix shows the AUC, several binary performance metrics, and the performance diagram for the VAE Long ASHA model in (a-c) respectively, for the six training data sets. Additional ML results in the form of confusion matrices of each model's performance on the RBM-generated sequence dataset are included in Figs 3, 2 and 4 in S3 Appendix.

References

1. Kingma DP, Welling M. Auto-Encoding Variational Bayes. CoRR. 2014;abs/1312.6114.
2. He K, Zhang X, Ren S, Sun J. Deep residual learning for image recognition. Proceedings of the IEEE Computer Society Conference on Computer Vision and Pattern Recognition. 2016;2016-December:770–778. doi:10.1109/CVPR.2016.90.
3. van der Spoel E, Rozing MP, Houwing-Duistermaat JJ, Eline Slagboom P, Beekman M, de Craen AJM, et al. Siamese Neural Networks for One-Shot Image Recognition. ICML - Deep Learning Workshop. 2015;7(11):956–963.
4. Müller R, Kornblith S, Hinton G. When does label smoothing help? Advances in Neural Information Processing Systems. 2019;32.
5. Miyato T, Kataoka T, Koyama M, Yoshida Y. Spectral normalization for generative adversarial networks. 6th International Conference on Learning Representations, ICLR 2018 - Conference Track Proceedings. 2018;.
6. Zhang H, Goodfellow I, Metaxas D, Odena A. Self-attention generative adversarial networks. 36th International Conference on Machine Learning, ICML 2019. 2019;2019-June:12744–12753.

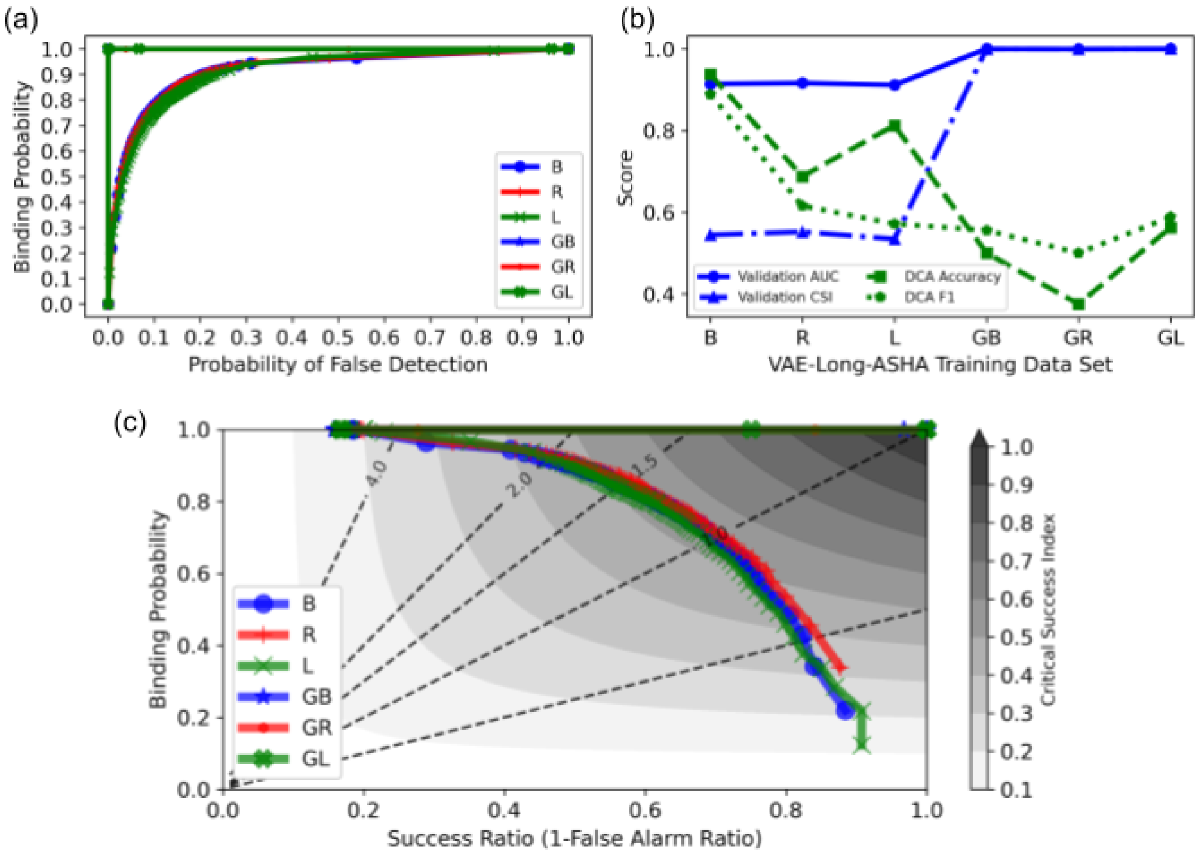

Figure 1: AUC (panel (a)), performance metrics (panel (b)), performance diagram (panel (c)) showing CSI for the VAE Long ASHA model.

Model	Validation Acc.	RBM Acc.	DCA Acc.	F1 mean
**AHSA Resnet Long**				
L	0.792	0.333	0.750	0.428
R	0.711	0.296	0.562	0.469
B	0.751	0.444	0.625	0.578
GL	0.999	0.778	0.375	0.718
GR	0.999	0.889	0.562	0.790
GB	0.998	0.778	0.438	0.729
**Bayes Resnet Long**				
L*	0.304	0.741	0.312	0.697
R*	0.281	0.704	0.312	0.683
B*	0.280	0.741	0.312	0.697
**AHSA Resnet Short**				
L	0.758	0.333	0.688	0.463
R	0.789	0.407	0.750	0.586
B	0.767	0.407	0.875	0.617
GL	0.998	0.778	0.438	0.729
GR	0.999	0.778	0.438	0.729
GB	0.999	0.852	0.562	0.777
**Bayes Resnet Short**				
L*	0.384	0.741	0.312	0.697
R	0.796	0.444	0.688	0.528
B	0.758	0.333	0.625	0.470
**AHSA VAE Long**				
L	0.828	0.333	0.812	0.416
R	0.837	0.333	0.625	0.492
B	0.819	0.333	0.875	0.510
GL	1.000	0.815	0.562	0.766
GR	1.000	0.778	0.500	0.741
GB	1.000	0.778	0.562	0.754
**Bayes VAE Long**				
L	0.804	0.307	0.688	0.401
R	0.838	0.407	0.688	0.505
B	0.829	0.296	0.750	0.450
**AHSA VAE Short**				
L	0.757	0.296	0.688	0.365
R	0.789	0.481	0.875	0.623
B	0.776	0.407	0.812	0.574
GL	1.000	0.741	0.562	0.739
GR	1.000	0.889	0.688	0.822
GB	1.000	0.889	0.562	0.790
**Bayes VAE Short**				
L	0.804	0.333	0.562	0.360
R	0.803	0.370	0.812	0.542
B	0.801	0.407	0.875	0.581
**PBT Siamese**				
L	0.687	0.458	0.662	0.300
R	0.598	0.491	0.600	0.391
B	0.643	0.467	0.508	0.314

Table 2: Accuracy Scores for all models trained on the Left Arm (L), Right Arm (R) Both Arms (B), Generated Left Arm (GL), Generated Right Arm (GR) or Generated Both Arms (GB) datasets. Models with a star(*) were optimized to minimize the validation set loss. Validation sets were taken as 10% of the training data, while the experimental datasets consisted of the 27 RBM generated sequences and the 16 DCA generated sequences.

Model	Validation Acc.	RBM Acc.	DCA Acc.	F1 Mean
**Single Tree**				
L	0.116	0.778	0.313	0.708
R	0.122	0.697	0.313	0.741
B	0.114	0.778	0.375	0.718
GL	0.999	0.704	0.813	0.764
GR	0.999	0.852	0.813	0.832
GB	0.885	0.889	0.625	0.781
**Random Forest**				
L	0.242	0.630	0.438	0.665
R	0.270	0.630	0.313	0.651
B	0.294	0.630	0.313	0.651
GL	0.950	0.741	0.375	0.684
GR	0.942	0.778	0.375	0.695
GB	0.939	0.778	0.438	0.706
**Gradient Boosted Forest**				
L	0.098	0.741	0.313	0.697
R	0.097	0.741	0.313	0.697
B	0.099	0.741	0.313	0.697
GL	0.091	0.741	0.313	0.697
GR	0.091	0.741	0.313	0.697
GB	0.167	0.741	0.313	0.697

Table 3: Accuracy Scores for single tree, random forest and gradient boosted forest trained on the Left (L), Right (R), Both (B), Generated Left Arm (GL), Generated Right Arm (GR) or Generated Both Arms (GB) datasets. Validation sets were taken as 20% of the training data, while the experimental dataset consisted of the 27 RBM generated sequences and the 16 DCA generated sequences.

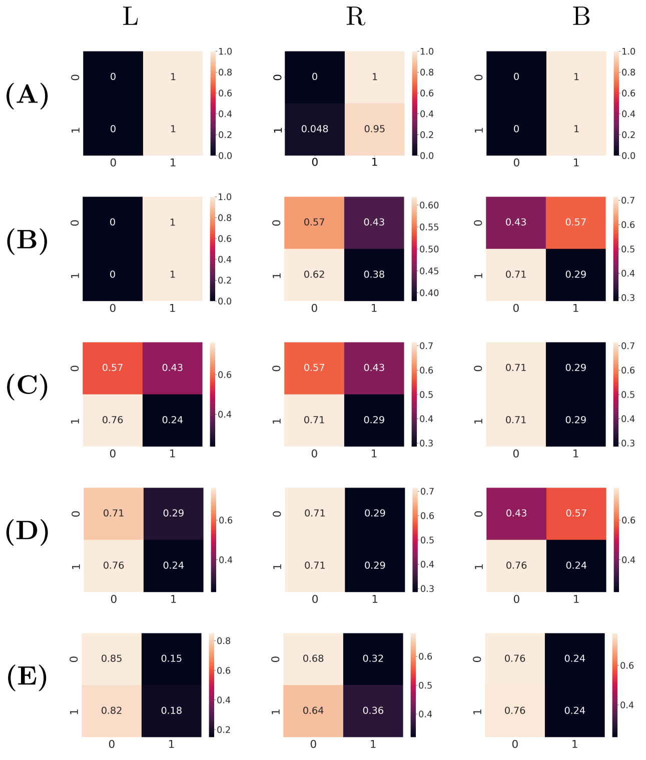

Figure 2: Confusion Matrices of trained loss-minimized or accuracy maximized bayesian optimized hyper-parameters on RBM generated dataset, (A) Long Resnet, (B) Short Resnet, (C) Short VAE, (D) Long VAE, and Population based training of Siamese Network (E). Predicted Label (0) nonbinder or (1) binder is shown on the x-axis with the true label being the y-axis.

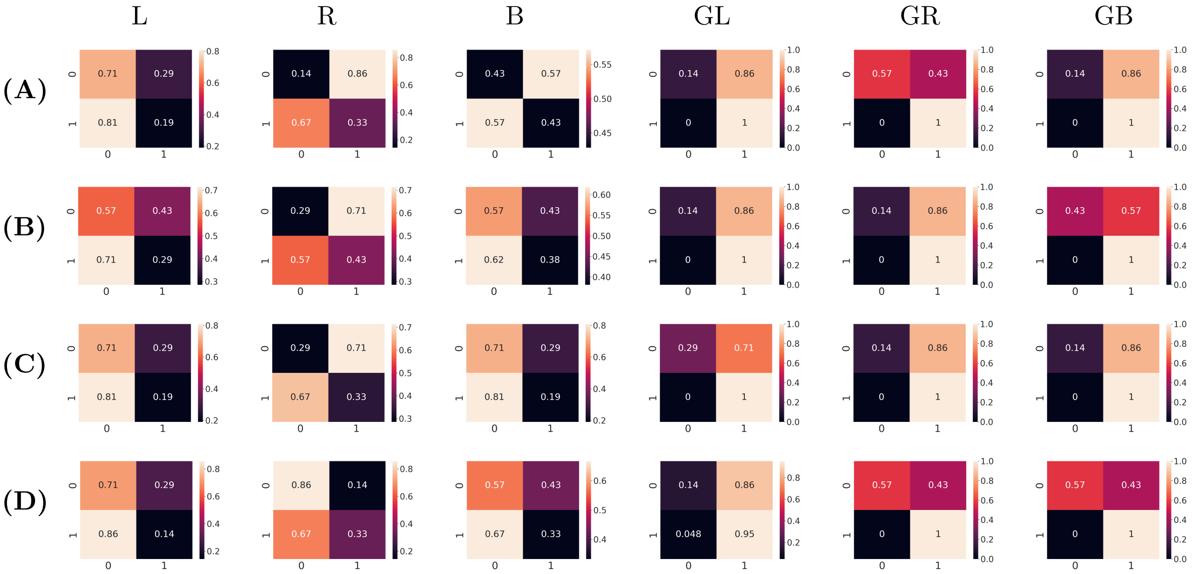

Figure 3: Confusion Matrices of accuracy maximized ASHA scheduler for hyper-parameter optimization using deep learning models: Long Resnet (A), Short Resnet(B), Long VAE (C) and Short VAE (D) on the RBM generated dataset. Predicted Label (0) nonbinder or (1) binder is shown on the x-axis with the true label being the y-axis.

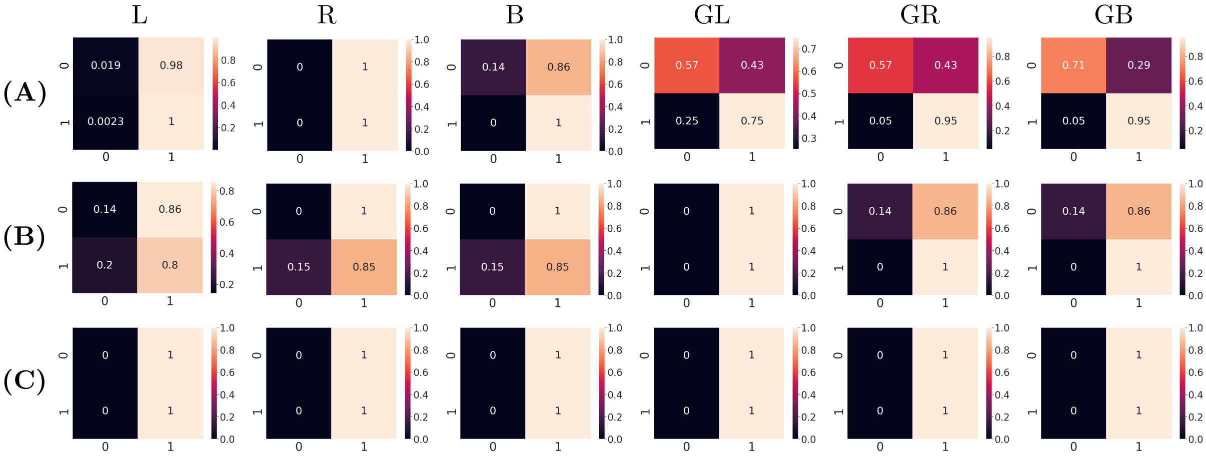

Figure 4: Confusion Matrices of traditional machine learning models: Single Tree, Random Forest and Gradient Boosted Forest on the RBM generated dataset. Predicted Label (0) nonbinder or (1) binder is shown on the x-axis with the true label being the y-axis.
