# Supplementary material for: Generative and interpretable machine learning for aptamer design and analysis of in vitro sequence selection
Source: PLoS Comput Biol. 2022 Sep 29;18(9):e1010561. doi: 10.1371/journal.pcbi.1010561 (PMC9553063; doi:10.1371/journal.pcbi.1010561)
Supplement: S4 Appendix — (PDF) [file pcbi.1010561.s026.pdf]

## Inference of sequencing error probability

We describe here our method for inferring the single-site sequencing error probability. The analysis here discussed is based on sequences from the left loop, collected at the last selection round (round 8). Repeating the analysis on the right loop provides analogous results.
Given a sequence $\sigma$ with high copy number $n_\sigma \gg 1$, the method uses as signal the number $\mu_\sigma$ of sequences that are at Hamming distance 1 from $\sigma$ and are never observed in the dataset. This number depends on the error rate, since a higher error rate is expected to cause more of these sequences to be detected. Since we consider only the left loop, sequences have a length $L = 20$ nt.
In Fig 1A in S4 Appendix we provide a representation of the sequence space around $\sigma =$ GGGTGATGTGTGGTAGGC , which is the sequences with highest copy number $n_\sigma = 8034$ in our dataset. The dots in a circle around $\sigma$ represent the $3 \times L = 60$ sequences that belong to the neighborhood of the main sequence $\mathcal{N}(\sigma)$, with color encoding their copy number. Some of these sequences are present $> 100$ times, and are unlikely to be an artifact of sequencing error. Other are present 1-2 times and can potentially be generated by sequencing errors. Finally, a number $\mu_\sigma = 12$ of sequences are absent in the sample (red crosses). These are mostly related to mutations removing one Guanine from the sequence, which might be related to a loss of fitness. While it is not possible to know with certainty whether one of the present neighbouring sequences with low copy-number was originated by sequencing error, the fact that some of these sequences are absent implies that $\sigma$ was never mis-read into these sequences. This information will be used in our inference. We start by selecting a number of sequences with high copy number. In Fig 1B in S4 Appendix we plot the number of sequences that have copy-number higher than a given threshold, as a function of the threshold. For our analysis we select as "peaks" all sequences with $n_\sigma > 1000$ (21 such sequences in the dataset). In Fig 1C in S4 Appendix we report the Hamming distance matrix for the selected sequences. As can be expected peaks tend to cluster together, with most of the peaks having at least one other peak in their neighbourhood. This can potentially increase the bias in our upper bound for the sequencing error probability. We will later introduce a correction to reduce this bias.

As a next step we define a probability for $\mu_\sigma$ as a function of the reading error probability. We call $\epsilon$ the probability of mis-reading a single nucleotide in the sequence. We consider this probability to be uniform along the sequence and on the real/read nucleotides, so that the probability of obtaining as outcome of sequencing $\sigma'$, one of the single-site mutations $\mathcal{N}(\sigma)$ of $\sigma$, when in reality reading $\sigma$ is:

$$P(\sigma'|\sigma) = p(\epsilon) = \frac{\epsilon}{3}(1-\epsilon)^{L-1}. \tag{1}$$

The real copy-number $\widetilde{n}_\sigma$ of $\sigma$ in the sample might be slightly different from the observed copy number $n_\sigma$, due to sequencing error. If we call $P(\sigma|\sigma) = (1-\epsilon)^L$ the probability of correctly reading $\sigma$, then for a small enough error, we can approximate

$$n_\sigma \simeq \widetilde{n}_\sigma P(\sigma|\sigma) + p(\epsilon) \sum_{\sigma' \in \mathcal{N}(\sigma)} \widetilde{n}_{\sigma'} \simeq \widetilde{n}_\sigma P(\sigma|\sigma). \tag{2}$$

For any given sequence $\sigma' \in \mathcal{N}(\sigma)$, the probability of never mis-reading $\sigma'$ when in reality sequencing $\sigma$ is given by:

$$P(n_{\sigma'} = 0) = (1 - p(\epsilon))^{\widetilde{n}_\sigma} = q(\epsilon, n_\sigma). \tag{3}$$

Finally, the probability that in the neighbourhood of $\sigma$ a number $\mu_\sigma$ of sequences are never observed, provided that in reality they were never present, is:

$$P(\mu_\sigma|n_\sigma, \epsilon) = \mathrm{Binom}\big[|\mathcal{N}(\sigma)|, q(\epsilon, n_\sigma)\big](\mu_\sigma) = \binom{|\mathcal{N}(\sigma)|}{\mu_\sigma} \left(q(\epsilon, n_\sigma)\right)^{\mu_\sigma} \left(1 - q(\epsilon, n_\sigma)\right)^{|\mathcal{N}(\sigma)| - \mu_\sigma}, \tag{4}$$

where $|\mathcal{N}(\sigma)| = 60$ is the size of the neighbourhood of $\sigma$. When writing this equation we are making a number of simplifications. On one hand we are considering that all sequences in $\mathcal{N}(\sigma)$ were originally absent in the sample. Moreover we are neglecting the probability that reads of these sequences might be generated from the sequencing of other sequences different from $\sigma$ (e.g. other peaks). All of these effects will bias our estimate, but the bias is always in the same direction, leading us to overestimate $\epsilon$. For this reason the result of the inference represents a reliable upper bound.
To reduce the bias we can remove from the total number of trials in the binomial the number of sequences that we are confident to be really present in the original sample. As a simple correction, we substitute the term $|\mathcal{N}(\sigma)| = 60$ in Eq (4) in S4 Appendix with $|\{\sigma' \in \mathcal{N}(\sigma) \text{ s.t. } n(\sigma') \leq 10\}|$, i.e. the number of sequences in the neighbourhood with no more than 10 counts. That is to say we consider all sequences

with more than 10 counts to be really present in the original sample. We perform the inference both with and without this correction (cf. Fig 1D in S4 Appendix).
At this point we can write the total log-likelihood of our data as a function of the error probability $\epsilon$ as:

$$\log \mathcal{L}(\text{data}|\epsilon) = \sum_{\sigma \in \text{peaks}} \log P(\mu_\sigma | n_\sigma, \epsilon) \ \propto \ \log \mathcal{L}(\epsilon|\text{data}), \tag{5}$$

where the inversion was operated using Bayes theorem with an uniform prior for $\epsilon$. In Fig 1D in S4 Appendix we display the behavior of the log-likelihood as a function of $\epsilon$ for the two cases, with and without correction. Numerical maximization of these functions yields values of $\epsilon^* \sim 10^{-3}$ as an upper bound for the error probability. To obtain a confidence interval on these bounds one can perform a Gaussian fit on the likelihood (i.e. a quadratic fit of the log-likelihood) around its maximum, and use the variance of the inferred Gaussian to obtain a confidence interval for the inferred value. In this case we obtain a standard deviation of the order of $4 \times 10^{-5}$.
In conclusion, we are confident that the single-site sequencing error probability in our dataset is smaller than $10^{-3}$.

## Estimation of number of sequencing error artifacts in the dataset

We can make use of the previously derived upper bound for $\epsilon$ to provide an upper bound for the number of unique sequences in our dataset that could be generated by sequencing error.
Since we expect double errors to be sufficiently rare in our dataset (for $\epsilon^* \sim 10^{-3}$ the probability of having more than 1 error is $\sim 2 \times 10^{-4}$), we can consider that in order to be an error, all the reads of a sequence $\sigma$ in our dataset must be generated by sequences in its neighbourhood $\mathcal{N}(\sigma)$, with the probability of mis-reading being equal to $p(\epsilon)$ (see Eq (1) in S4 Appendix). For each sequence we define:

$$N_\sigma = \sum_{\sigma' \in \mathcal{N}(\sigma)} n_{\sigma'}. \tag{6}$$

This is the total number of sequences in the neighborhood of $\sigma$. Because of sequencing error the real number might be slightly higher, and as done for $n_\sigma$ one can introduce the correction $\widetilde{N}_\sigma = N_\sigma/(1-\epsilon)^L$. We can take as an upper bound for the probability of $\sigma$ to be an artifact of sequencing error, the probability that by reading $\widetilde{N}_\sigma$ sequences in the neighbourhood of $\sigma$, we read $\sigma$ a number of time equal or greater than the observed copy-number $n_\sigma$:

$$P(n_{\text{err}} \geq n_\sigma) = \pi(\sigma, \epsilon) = \sum_{k=n_\sigma}^{\infty} \text{Binom}[N_\sigma, p(\epsilon)](k) \tag{7}$$

We numerically evaluate this probability for every sequence $\sigma$. The value of $N_\sigma$ is efficiently computed by generating all possible single mutations $\sigma' \in \mathcal{N}(\sigma)$, and quickly recovering their copy-number using a hash table.
In Fig 2 in S4 Appendix we report the distribution of $\pi(\sigma, \epsilon^* = 10^3)$ for all of the sequences in our dataset. For the great majority of the sequences this probability is very low. From the procedure we employ it follows that sequences with the highest probability of being errors are ones that have very low $n_\sigma$ and with a highly populated neighbourhood (high $N_\sigma$). By treating the reality of each unique sequence as a Bernoulli random variable, the mean and variance for the total number of unique sequences that we expect to be an artifact of sequencing error can be expressed as:

$$E[N_{err}] = \sum_\sigma \pi(\sigma, \epsilon^*) \quad Var[N_{err}] = \sum_\sigma \pi(\sigma, \epsilon^*)(1 - \pi(\sigma, \epsilon^*)) \tag{8}$$

This gives an estimate $N_{err} \sim 941 \pm 28$. Since our dataset is composed of roughly $2 \times 10^5$ unique sequences this upper bound represents only 0.5% of the total dataset, and it is not expect to meaningfully impact the training of our models.

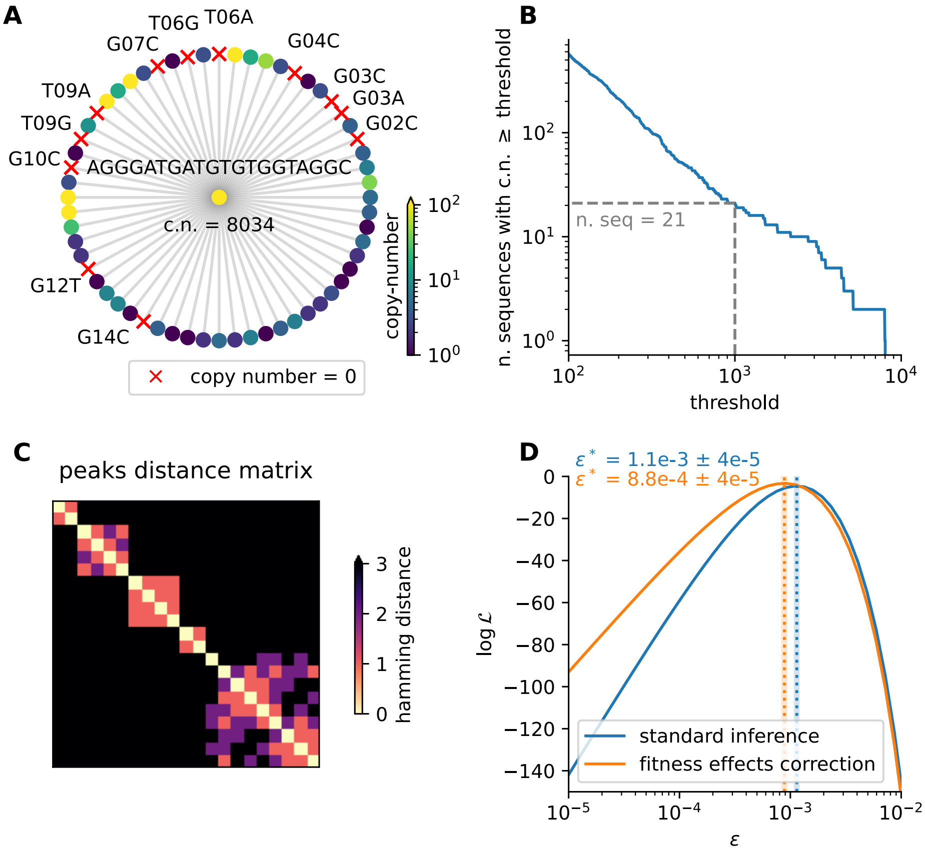


Figure 1: inference of an upper bound for the sequencing error probability in our sample, using sequences from the left loop in the 8th round.

Panel A: example of sequence space around the most abundant sequence in the dataset. The main sequence is represented as a dot in the center, and the full DNA sequence and copy number (c.n.) are reported. Dots around it represent sequences at hamming distance 1, with color encoding their copy number. Sequences that were never detected in the sample are indicated with red crosses. For these sequences we report the difference from the main sequence as a triplet (original nucleotide, position, substituted nucleotide). Notice how some of the neighboring sequences have high copy number, indicating probable fitness effects. Most of the non-detected sequences are associated with removal of a guanine, which might decrease binding affinity.

Panel B: number of sequences with copy-number greater than a given threshold. For our analysis we select only sequences with c.n. $\geq 1000$ (21 such sequences in the dataset). These sequences are referred to as "peaks" in the analysis.

Panel C: relative Hamming distance between peak sequences. High-copy-number sequences tend to cluster together. This can cause a less precise estimation of the inferred sequencing error upper bound, since the neighbourhood of a peak can be populated by other high-fitness sequences. To correct for this we introduce a correction that removes sequences with c.n. $> 10$ from the expression of the likelihood.

Panel D: log-likelihood of the single-site sequencing error probability $\epsilon$. The inference was performed in two ways: either using the standard approach (blue) or introducing the correction for fitness effects (orange). In each case we mark the inferred value $\epsilon^*$ with vertical dotted lines. The thin shaded area represent the confidence interval, that was derived through a Gaussian fit of the log-likelihood in proximity of its maximum.

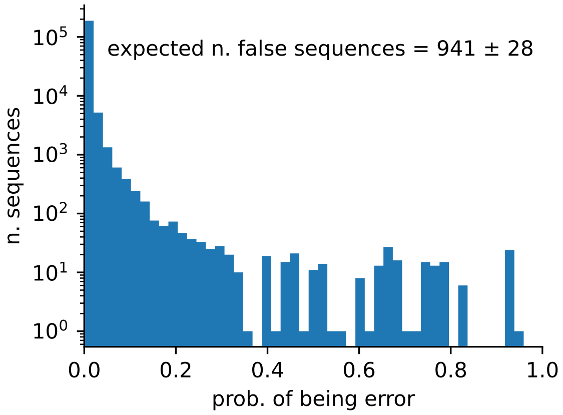


Figure 2: Distribution of inferred single-sequence error probabilities. For each sequence in the considered dataset (round 8, left loop) we infer the probability of being an artifact of sequencing error. In the inference the single-site error probability was set equal to the upper bound $\epsilon^* = 10^{-3}$. The vast majority of sequence have a zero or low probability of being sequencing error artifacts. From this distribution one can evaluate the mean and standard deviation of the total number of artifacts. This gives an upper bound of $N_{err} = 941 \pm 28$, which corresponds to a 0.5% of the total number of unique sequences in the dataset considered.
