# Supplementary material for: Generative and interpretable machine learning for aptamer design and analysis of in vitro sequence selection
Source: PLoS Comput Biol. 2022 Sep 29;18(9):e1010561. doi: 10.1371/journal.pcbi.1010561 (PMC9553063; doi:10.1371/journal.pcbi.1010561)
Supplement: S5 Appendix — (PDF) [file pcbi.1010561.s027.pdf]

Comparison with Direct Coupling Analysis

Direct Coupling Analysis (DCA) is a method of analysis originally used for contact prediction in proteins from sequence alignments of homologues. The basis of this method is that the homologue alignments have the same general native state to carry out their function. Despite their differences in sequence, all homologues will have similar inter-domain contacts. To maintain these contacts, detrimental single site mutations must be offset by compensatory mutations in other parts of the sequence. DCA is a maximum-entropy method, where the model parameters are fixed so that the one- and two-point correlations along the sequences are fixed to those observed in the training homologue-sequence aligment. The sequence probability is given in Eq (1) in S5 Appendix and is dependent on the learned single position parameters (h) and pairwise interactions (J_{ij}) of the multiple sequence alignment.

$$P(\sigma) = \frac{1}{Z} \exp\left(\sum_{i=1}^{L} h_i(\sigma_i) + \sum_{1 \leq i < j \leq L} J_{ij}(\sigma_i, \sigma_j) \right). \tag{1}$$

Similar to the protein case, we applied DCA on our aligned DNA aptamer dataset to approximate the aptamer sequence space with the learned single site and pairwise correlations. Sequences unobserved in the original dataset were generated from the learned parameters and tested experimentally.

DCA Training

The training set used for DCA analysis was a subset (90%) of sequences with copy number > 1 from the 8th round of selection. Rather than separate the arms of each nanotile, the DCA model was trained with on 40 nt long sequences containing both arms. The normalization constant Z is difficult to calculate, so we use psuedolikelihood maximzation DCA (plmDCA) [1] to obtain local fields (h_i) and pairwise coupling ($J_i j$) for the model, given the aligned aptamer dataset. Monte Carlo sampling was applied across a range of temperatures and mutation steps to sample from the learned parameters. In total $2{*}10^9$ sequences were sampled, and from those 16 sequences shown in S1 Table were selected for experimental validation of the model.

DCA Sequence Selection

From the generated sequences, we wanted to find not only novel binders but also verify the learned model parameters. Sequences are scored according to the sum of their single position and pairwise parameters. A sequence's higher score indicates it is more likely to bind while a lower score indicates it is less to bind. Predicted binders (sequences d1, d2, d3, d4, d5, d11, d12, d13) were selected from the MC-generated sequences by having the highest score while being at least 3 mutations away from anything observed in the entirety of the 8th round of sequencing data. Two predicted nonbinders (d6, d14) were selected for having the lowest score within 2 mutations of the dataset. Rationally designed binders (d7, d8, d9, d15, d16, d17) were generated by randomly selecting a good and bad binder from the original dataset and altering them to either have the highest or lowest score possible by exhaustively calculating the entire sequence space within 3 mutations and finding the variant with the highest or lowest score. Model parameters used to generate all sequences are shown in Fig 1 in S5 Appendix.

DCA Gel Shift Assay

Sequences generated using the plmDCA model were tested experimentally for their ability to bind Thrombin. Binding sequences formed a clear protein / stem-loop band. Sequences were tested the same way as done for the RBM-generated sequences in the main text. S18 Fig shows the experimental results of a gel shift assay for the plmDCA generated sequences.

DCA Binding Site Assay

Thrombin binding sequences generated via plmDCA (d11, d12, d13, d14, d16) were tested against known binders 5' 6FAM labeled ThA and ThD to determine their binding site as described in the main text. Table 1 in S5 Appendix contains the results and S18 Fig shows the gel results.

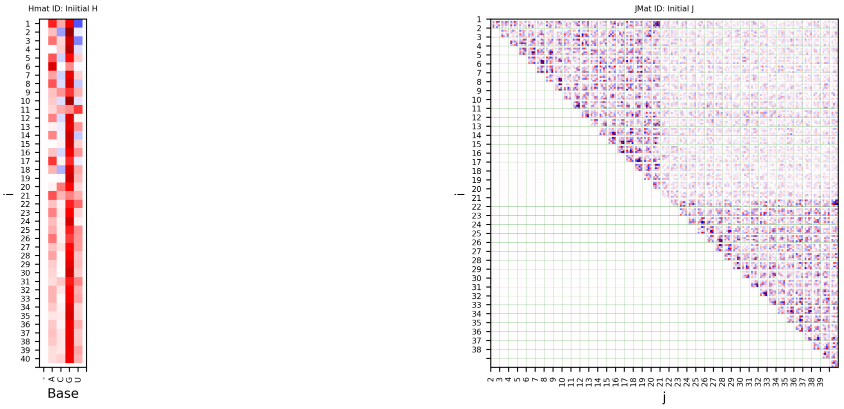

Figure 1: Single position (H) and pairwise correlations (J_{ij}) learned by the plmDCA model and used in both sampling and sequence selection.

Label	Sequence	Binding Site
d11	GTAGGATGGGTGGGGTGGGA	exosite I
d12	GTAGGATGGGTAGGGTGGTA	exosite I
d13	CTAGGTTGGGTAGGGTGGTG	exosite I
d14	CTAGCATGGGTAGGGTGGTG	exosite I
d16	TTGGGTGGTGTAGGTTGGCG	exosite I

Table 1: Exosite prediction of DCA sequences that bound thrombin from our gel shift assays.

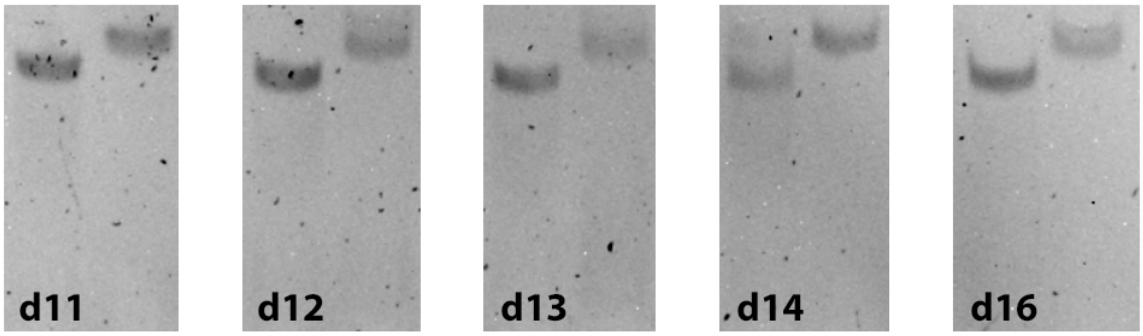

Figure 2: Binding site assay using the same method discussed in the main text. Lane 1 is the result of the preincubated strand exposed to exosite-II binder ThA and lane 2 is the preincubated strand exposed to exosite-I binder ThD.

DCA Results

The weak pairwise correlations seen in the top right corner of the pairwise correlation matrix (J_{ij}) confirm the lack of correlation between the two arms of each nanotile. The plmDCA method did see limited success in generating novel binders (d11, d12, d13, d16) from the right loop sequences but no success in generating binding left loop sequences (d1, d2, d3, d4, d5) (Fig 2 in S5 Appendix).

We tried also to train a DCA using the same algorithm we used for the RBM models to obtain the model parameters, i.e. the persistent contrastive divergence algorithm. Moreover, building on the results obtained with our RBM models, we decided to use all the available sequences to train the BM model, neglecting the counts. Then we compared, for the obtained DCA model trained with single-loop sequences at round 8, the log-likelihood assigned by the DCA with the one assigned by an RBM trained on the same data. The resulting plot is given in Fig 3 in S5 Appendix, and this test gave a very good linear correlation between the log-likleihoods of the two models (slope of the linear fit: 1.09; R^2 score: 0.97), suggesting that the DCA model trained with persistent contrastive divergence has superior generalization capabilities with respect to plmDCA models. This result is compatible with what observed in [2].

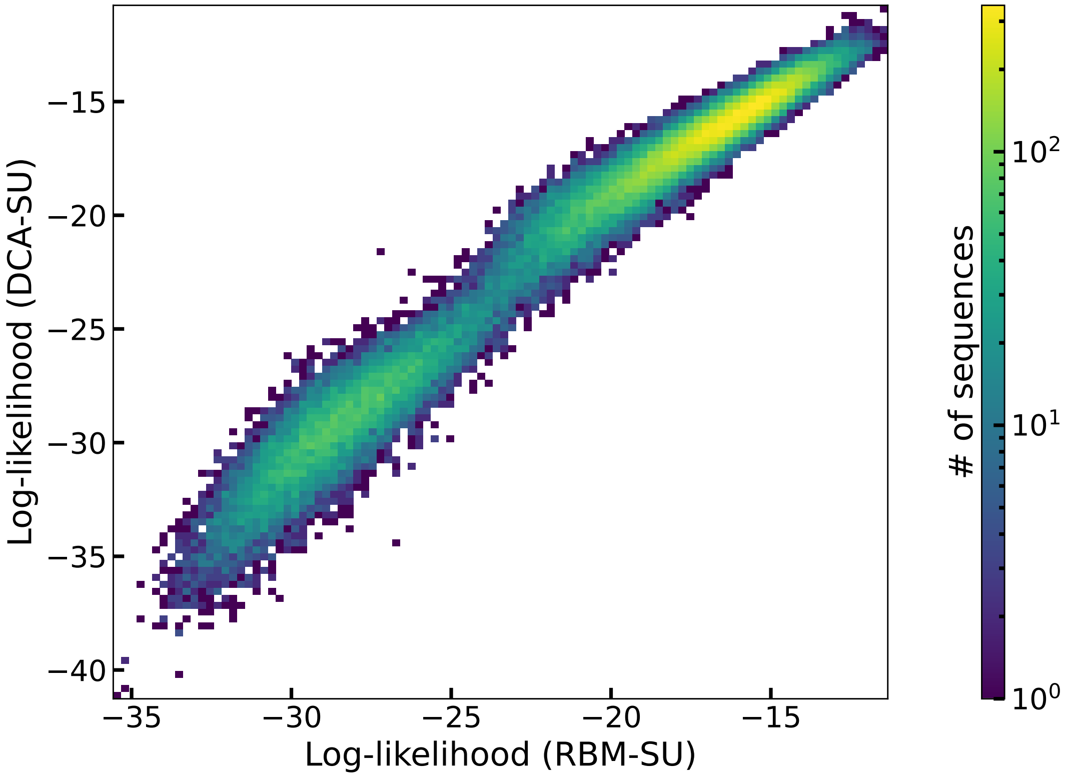

Figure 3: Log-likelihood of all unique single-loop aptamers observed at round 6, as computed by a DCA and an RBM model trained through persistent contrastive divergence. The corresponding linear fit resulted in a slope of 1.09 and an R^2 of 0.97.

References

1. Ekeberg M, Hartonen T, Aurell E. Fast pseudolikelihood maximization for direct-coupling analysis of protein structure from many homologous amino-acid sequences. Journal of Computational Physics. 2014;276:341–356. doi:10.1016/j.jcp.2014.07.024.
2. Barton JP, De Leonardis E, Coucke A, Cocco S. ACE: adaptive cluster expansion for maximum entropy graphical model inference. Bioinformatics. 2016;32(20):3089–3097. doi:10.1093/bioinformatics/btw328.
